# Rapid Microfluidic Drug Sensitivity Testing Within 5 Days Using Minimal Clinical Tumor Samples

**DOI:** 10.1002/advs.202511065

**Published:** 2025-12-08

**Authors:** Yi‐Xue Chen, Yi Zhang, Yu‐Jie Yan, Meng‐Ting Zhang, Jian‐Bo Chen, Yi‐Rong Jiang, Jie Wu, Hui‐Feng Wang, Jian‐Zhang Pan, Zhi‐Gang Chen, Jian Huang, Qun Fang

**Affiliations:** ^1^ Institute of Microanalytical Systems Department of Chemistry Zhejiang University Hangzhou 310058 China; ^2^ Department of Breast Surgery (Surgical Oncology) Second Affiliated Hospital Zhejiang University School of Medicine Hangzhou 310009 China; ^3^ Single‐cell Proteomics Research Center ZJU‐Hangzhou Global Scientific and Technological Innovation Center Hangzhou 311200 China; ^4^ Key Laboratory for Biomedical Engineering of Ministry of Education Cancer Center Zhejiang University Hangzhou 310007 China; ^5^ Key Laboratory of Tumor Microenvironment and Immune Therapy of Zhejiang Province Hangzhou 310052 China; ^6^ Cancer Centre Zhejiang University Hangzhou Zhejiang 310058 China

**Keywords:** 3D cell culture, high‐throughput drug screening, microfluidics, organs‐on‐a‐chip, personalized therapy

## Abstract

Rapid screening of personalized drugs based on patients’ primary cell samples can provide precise and timely treatment guidance for clinical oncology patients. However, this goal faces great challenges due to the scarce clinical samples and large sample consumption, and long experimental time required by current drug screening methods. Here, a rapid, high‐throughput microfluidic drug sensitivity testing system capable of accomplishing single and combination drug screening of multiple antitumor drugs in 5 days is established with minimal amounts of clinical primary tumor samples, avoiding the need for cell pre‐expansion and preserving the tumor heterogeneity. An airflow‐impacting approach is developed to fabricate nanoliter‐scale microcavity arrays with ultra‐smooth microcavity surfaces, with which rapid formation and 3D culture of tumor cell spheroids from small numbers of cell samples, as well as the subsequent high‐throughput drug sensitivity testing can be achieved within 5 days. This applies the system in rapid drug sensitivity testing on primary samples from 21 clinical breast cancer patients to quantify the responses of patient‐derived cells to chemotherapy and endocrine drugs under both the mono‐drug and combinational‐drug treatment modes.

## Graphical abstract

This study develops the air‐punched fabrication approach for nanoliter‐scale microcavity arrays with ultra‐smooth microcavity surfaces, with which rapid formation and 3D culture of tumor cell spheroids from small numbers of cell samples, as well as high‐throughput drug sensitivity testing can be achieved within 5 days. The system is applied in rapid, high‐throughput drug sensitivity testing on primary cell samples from 21 clinical breast cancer patients to quantify the responses to chemotherapy drugs and endocrine drugs.

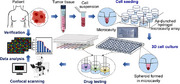

## 1 Introduction

Cancer is one of the leading causes of human death in the world. According to the data of World Health Organization, nearly 10 million people died of cancer in 2020.^[^ ^1^ ^]^ Standard‐of‐care cancer therapy includes surgery, radiation, chemotherapy, immunotherapy, targeted therapy and endocrine therapy. Drug therapy is one of the main therapeutic options for cancer treatment. However, personalized differences among patients lead to enhanced heterogeneity among tumors in their bodies, which is clinically manifested as different resistance of different patients to the same drug regimen.^[^ ^2^ ^]^ Preclinical drug testing geared toward individualized therapy has the potential to obtain information on the sensitivity of clinical patients to different drugs^[^ ^3^ ^]^ and can provide effective data base for patient treatment, thereby improving patient survival.

One popular approach for preclinical individualized drug testing is to use patient‐derived cells in 2D culture. 2D culture is the most commonly used preclinical culture method in vitro due to easy operation, low cost, good reproducibility, and suitability for high‐throughput screening.^[^ ^4^ ^]^ However, there are limitations of the traditional 2D culture models in representing the real microenvironment in terms of structure, mechanical properties, intercellular communication, and drug response.^[^ ^5^ ^]^ It has been reported that some drug response results obtained in 2D culture models are inconsistent with those of the clinical studies, which can affect the accuracy of clinical decisions.^[^ ^6^, ^7^ ^]^ Therefore, preclinical in vitro drug test models based on 3D culture are gradually being studied more and more.^[^ ^8^ ^]^ Compared with 2D culture models, 3D culture models are closer to the human body in terms of cell phenotype, microenvironment, gene and protein expression, differentiation, and drug resistance, which can better simulate the real tissue microenvironment.^[^ ^9^, ^10^ ^]^ The current methods for constructing 3D cell culture models include hanging drop,^[^ ^11^ ^]^ non‐adhesive substrate‐coated culture surfaces,^[^ ^12^ ^]^ ultralow attachment plates,^[^ ^13^ ^]^ spinner flasks,^[^ ^14^ ^]^ rotating bioreactors,^[^ ^15^ ^]^ magnetic levitation,^[^ ^16^ ^]^ and gel matrix encapsulation.^[^ ^17^ ^]^ In the above methods, cell aggregation to form spheroid is one of the main features in constructing 3D culture models, within which the distribution of oxygen and nutrients as well as the intercellular connections are similar to those of tissues in human body.^[^ ^18^ ^]^ In most 3D culture models, the diameters of single cell spheroids usually vary from tens of micrometers to several millimeters, containing 10^3^–10^8^ cells in each spheroid.^[^ ^19^ ^]^ Generally, the generation of cell spheroids takes ≈ 1 week, while the construction of patient‐derived tumor organoid takes longer, typically 1–6 months. However, in order to make personalized clinical decisions in time, it is necessary to obtain drug sensitivity information of patients within 1‐2 weeks after acquiring the patients’ cell samples. It is difficult for using the current cell expansion culture method to meet the critical need for rapid drug therapy in clinical treatment. Notably, patient‐derived primary tumor cells retain the complex microenvironment in vivo and patients’ genetic information. Tumor primary cells that are used directly for drug testing without expansion culture can provide high fidelity, and this approach can reduce time cost while simultaneously enhance the accuracy of drug sensitivity testing results in preclinical drug prediction. Currently, most clinical primary tumor samples from non‐surgical or pre‐surgical patients are obtained through procedures such as biopsy and endoscopic sampling. In clinical practice, ≈78% of tumor diagnoses rely on biopsy, which are of critical importance for timely disease identification and therapeutic decision‐making. However, the limited amount of these samples remains a major constraint in performing patient‐individualized drug sensitivity testing. The total number of cells obtained from a single biopsy is usually limited to 10^4^–10^5^ cells. After a portion of the biopsy samples are applied to diagnostic pathology, there are even fewer remaining samples available for therapeutic drug sensitivity testing. Using conventional multiwell plate‐based drug sensitivity testing methods, a single drug‐dose condition typically requires ≈2 × 10^3^–10^4^ cells.^[^ ^20^, ^21^ ^]^ Considering that at least six orders of magnitude of drug concentrations need to be measured with at least three repetitions of each concentration point, the available biopsy samples can usually support testing of only 1‐2 drugs at a single dose level, which is far from meeting the needs of individualized drug sensitivity screening usually requiring screening more than five different drugs and various combinations of these drugs.^[^ ^21^ ^]^

**Figure 1:**
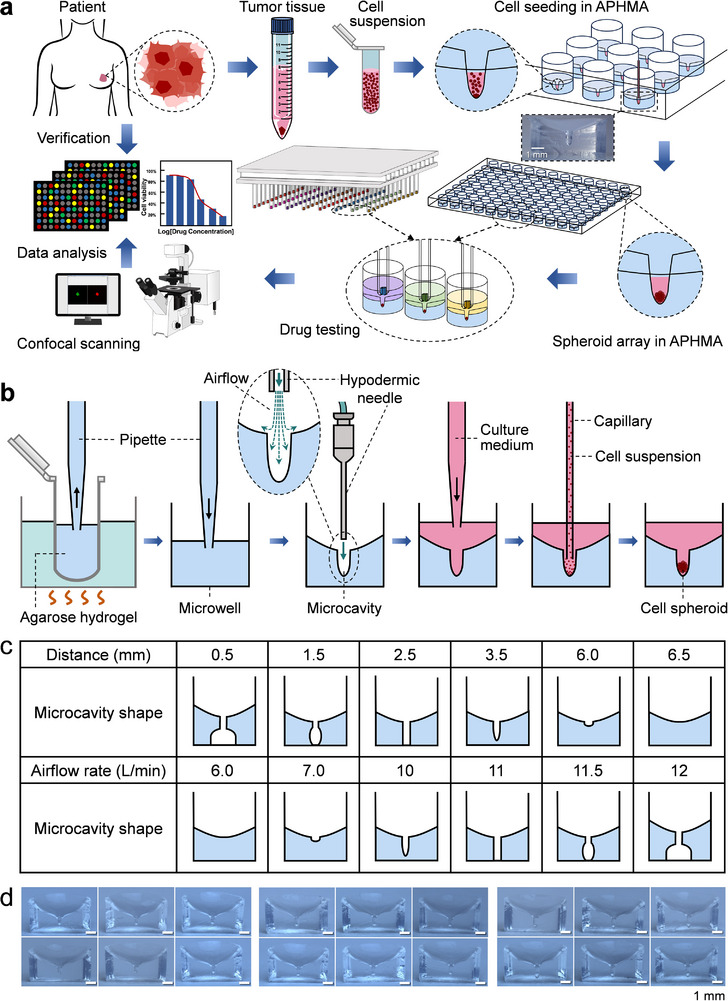
a) Schematic diagram of the workflow for rapid 3D culture of ultra‐micro‐scale patient‐derived primary cells and drug sensitivity tests based on APHMA platform. Scale bar, 1 mm. b) Schematic diagram of the preparation of APHMA and the formation of spheroids. c) Effects of airflow rate and probe tip‐to‐hydrogel surface distance on the shape of microcavity arrays. d) Photographs of microcavity arrays at airflow rates of 9.7, 10.2, and 10.7 L min^−1^, respectively. Scale bars, 1 mm.

To overcome the above challenges, we developed an ultra‐smooth surface‐based microcavity array platform for ultra‐micro samples of patient‐derived primary cells for rapid 3D spheroid culture and drug sensitivity testing (**Figure** **1**a), with which drug effect results can be obtained within 5 days after sampling. We developed the microfluidic airflow impact method to fabricate nanoliter‐scale U‐shaped air‐punched hydrogel microcavity array (APHMA) with ultra‐smooth microcavity surfaces, capable of achieving rapid 3D cell‐spheroid formation of minimal samples as small number as 10 cells. Additionally, we also developed a micropillar array matched with the microcavity array system for efficient and high‐throughput drug delivery and screening. We applied the present system to actual clinical drug sensitivity testing in breast cancer patients. Multiple drug response results including mono‐drug and combination drug treatments in chemotherapy and endocrine therapy were obtained within 5 days.

## 2 Results

### 2.1 Development of the APHMA Platform

Currently, there are two main methods for performing cell spheroid culture.^[^ ^22^, ^23^ ^]^ One method is based on the first migration and the subsequent aggregation of dispersed cells to achieve the formation of cell spheroids.^[^ ^24^ ^]^ The other method employs the first physical aggregation of cells by external forces to induce the ultimate formation of cell spheroids.^[^ ^25^ ^]^ The former method mainly relies on the interaction of the cells to achieve their aggregation with each other, which usually needs longer times of 1‐3 weeks to obtain cell spheroids.^[^ ^26^ ^]^ The latter method usually requires the use of the special devices with microstructures produced by microfabrication techniques, such as micropattern,^[^ ^27^ ^]^ matrix encapsulation,^[^ ^13^ ^]^ hanging drop,^[^ ^28^ ^]^ and magnetic levitation method,^[^ ^29^ ^]^ which could significantly shorten the formation time of cell spheroids. A commonly‐used microfabrication method is to use the replica molding technique^[^ ^30^ ^]^ to fabricate the cell sample containers that can facilitate cell aggregation. These containers need to have smooth sidewall surfaces to facilitate cell aggregation, as rough surfaces will trap some cells causing cell dispersion and prolonged spheroid formation time. At the preliminary stage of this work, we tried to prepare agarose hydrogel microcavity arrays using the replica molding method with the copper molds. However, the surface and structure of these hydrogel microcavities were easily damaged during the demolding process, and in actual cell experiments, we observed that 10%‐50% of the cells added to the microcavities were trapped on the sidewalls of the microcavity. Among these trapped cells, ≈ 30% of them settled to the bottom of the microcavities after 3‐day culture, while there were still more than 70% of these cells did not settle to the microcavity bottom. Although we tried to adopt measures to treat the surface of the molds, such as polishing and surface coating, they had no significant effect on the resolution of the cell trapping phenomenon.

Here, we developed a replica molding‐based approach for the fabrication of agarose hydrogel microcavity arrays using the non‐contact airflow impaction effect instead of a solid mold, to produce microcavities with ultra‐smooth surfaces for facilitating the formation of cell spheroids and 3D cell culture. Most hydrogels used in cell culture have temperature‐sensitive properties, i.e., the solid and liquid states of the hydrogels change with temperature. At the preliminary stage of the work, we occasionally observed that when a high‐speed airflow from a small‐diameter tube impacted a hydrogel solution at a relatively high temperature, a U‐shaped microcavity would be generated in the hydrogel solution due to the impact of the airflow. With the continuous airflow impacting, the cooling effect of the airflow at a relatively lower temperature would make the hydrogel microcavity be solidified into a solid microcavity shortly. Since no solid mold was used to direct contact with the hydrogel solution, instead a continuous airflow was used to contact with (impact) the hydrogel solution, this resulted in the formation of the hydrogel microcavities with extremely smooth sidewalls. Figure 1b shows a typical process of the formation of hydrogel microcavity array formed by the airflow punching approach. Based on this airflow punching molding principle, we constructed a system (see details later in this section) for fabricating hydrogel microcavity arrays in 96‐well plates, which was capable of utilizing a continuous, stable airflow came from a stainless‐steel probe, to sequentially impact the agarose hydrogel solution pre‐loaded in each well of a 96‐well plate under the control of an automated movement system, forming the array of hydrogel microcavities (Movie S1, Supporting Information). After the hydrogel microcavity array was fabricated, we first used HeLa cells as model samples to test the smoothness of the microcavity sidewall. After tens of HeLa cells were added into a microcavity, all of these cells settled to the bottom of the microcavity within 5 min without the aid of any external force, and no stuck cells on the sidewall were observed (Movie S2, Supporting Information). In contrast, as previously reported methods, it generally takes 60‐90 min or even longer for cell aggregates to settle in conventional 96‐well plates or droplets.^[^ ^12^, ^31^ ^]^

We investigated and optimized various factors affecting the APHMA formation, including airflow rate, distance between the stainless‐steel probe tip end and the surface of the hydrogel solution, temperature of the hydrogel solution, duration of airflow impacting in a single microcavity, and the interval time of the airflow impacting between adjacent microcavities. During the fabrication of the microcavity arrays, we observed that the main factors affecting the shape of the microcavity arrays were the airflow rate and the distance between the stainless‐steel probe tip end and the surface of the hydrogel solution (i.e., probe tip‐to‐hydrogel surface distance). We tested the effects of these two factors on the formation of the microcavity arrays. When the airflow rate was less than 6.0 L min^−1^ and the probe tip‐to‐hydrogel surface distance was more than 6.5 mm, the U‐shaped microcavity structure could not be formed due to the insufficient impact of the airflow on the hydrogel. The U‐shaped microcavity structure could also not be formed when the airflow rate was more than 10.9 L min^−1^ and the probe tip‐to‐hydrogel surface distance was less than 2.1 mm, which would cause a strong oscillation of the hydrogel solution by the excessive impact of the airflow, as well as failing to form stable U‐shaped microcavity structures. Between these two boundary conditions, we observe that both the airflow rate and different probe tip‐to‐hydrogel surface distance could result in different configurations of microcavities, as shown in Figure 1c. Considering for the convenience of the cell aggregation, culture and drug experiments, we chose microcavities with appropriate depth of U‐shaped structure for the subsequent experiments. We further tested the consistency of the system in fabricating microcavities. Figure 1d shows typical images of microcavities formed at airflow rates of 9.7, 10.2, and 10.7 L min^−1^, respectively. Under these conditions, good consistency could be obtained in the hydrogel microcavity arrays. Finally, we chose the conditions to form the U‐shaped microcavity arrays at an airflow rate of 10.2 L min^−1^ and a probe tip‐to‐hydrogel surface distance of 3 mm.

### 2.2 3D Cell Culture and Spheroid Formation of Multiple Tumor Cell Lines in APHMA

**Figure 2:**
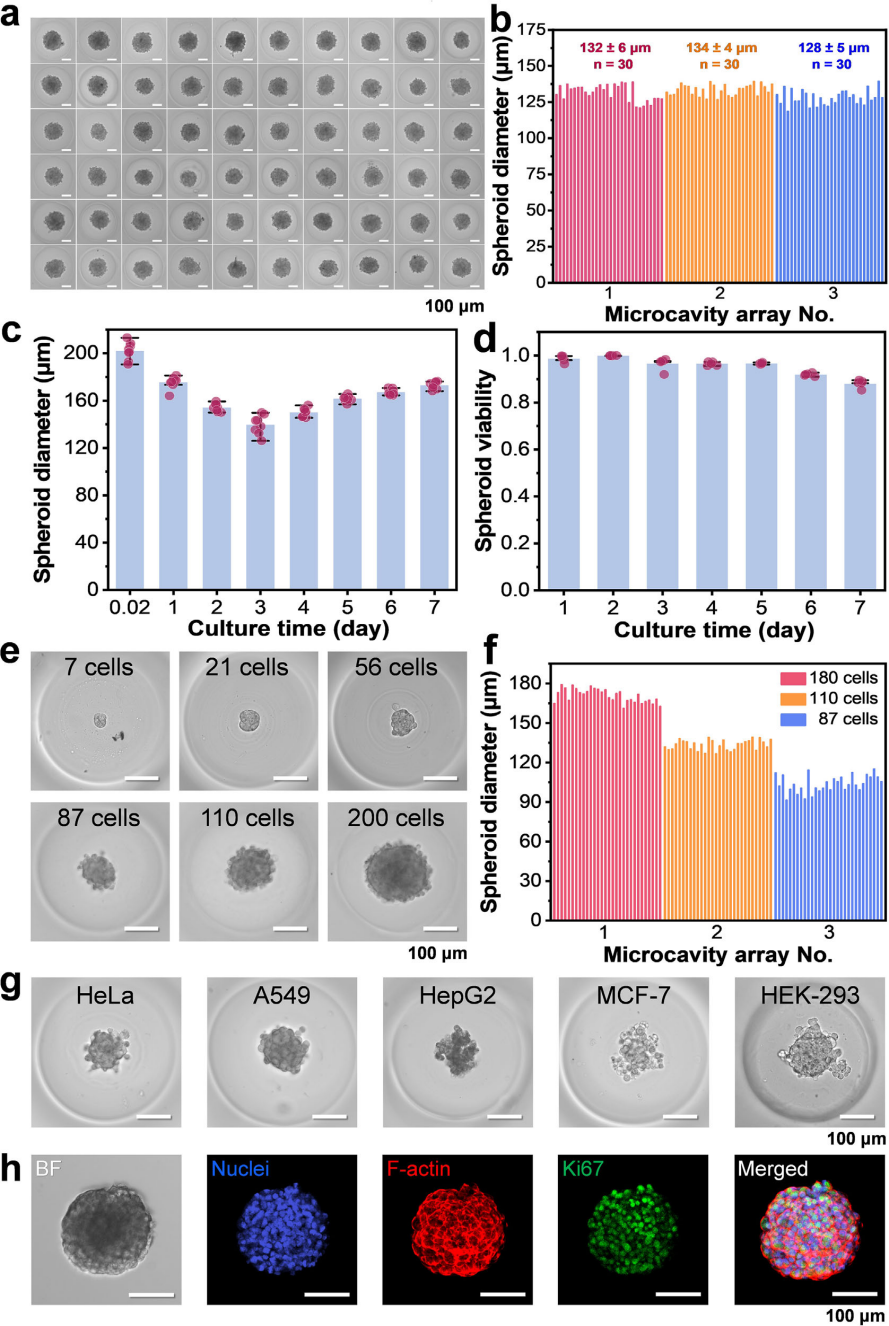
Tumor spheroids production in APHMA. a) The generation of HeLa 3D cell spheroids in a microcavity array after 72 h with an average cell seeding number of 180 cells in each microcavity. Scale bar, 100 µm. b) Distributions of HeLa spheroids diameters in three different APHMAs with 110 cells after 72‐h culture (n = 30). c) Distributions of HeLa spheroids diameters in an APHMA with 8 microcavities during 7‐d culture. d) Distributions of HeLa spheroids viability in an APHMA with 5 microcavities during 7‐d culture. e) The spheroid generation with different initial cell densities after 72 h. Scale bars, 100 µm. f) Distributions of spheroids diameters in APHMA with different average cell seeding numbers of 87, 110 and 180 in each microcavity after 72‐h culture. g) Generation of cell spheroids of HeLa cells, A549 cells, HepG2 cells, MCF‐7 cells and HEK‐293 cells in APHMA after 72 h. Scale bars, 100 µm. h) Bright field (BF) and immunofluorescence staining images of the spheroids with average cell seeding numbers of 110 cells after 7‐d culture, stained by Hoechst 33342 (blue), Alexa Fluor 555 Phalloidin (red) and Ki67 rabbit monoclonal antibody (green). The right‐most image shows the merged fluorescence image of the left three fluorescence images. Scale bars, 100 µm.

Under the optimal formation conditions for hydrogel microcavity arrays, different types of tumor cell lines were tested for validating the feasibility of 3D spheroid culture in APHMA. The seeding of cells in APHMA was automatically performed using the sequential operation droplet array (SODA) system previously developed by authors’ group.^[^ ^32^, ^33^ ^]^ First, 300 nL cell suspension with 110 density was sequentially added to each microcavity by the SODA system. The cells were cultured under appropriate culture conditions to form spheroid arrays and were observed and evaluated using the microscope. To assess the precision and accuracy of the system for cell seeding, equal volumes (300 nL) of cell suspensions at different cell densities were sequentially dispensed into individual wells of a 384‐well plate (n = 10), followed by counting the number of cells in each well. The results showed that when the expected average numbers of cells per well were 20, 80, and 150, the relative standard deviations (RSDs) were 9.4%, 3.7%, and 7.3% (n = 10), respectively, and the mean relative errors (MREs) were 8.5%, 3.0%, and 6.4% (n = 10), respectively (Figure S1, Supporting Information), demonstrating the favorable precision and accuracy of the system in cell seeding with different cell seeding numbers. We first used HeLa cells to test the APHMA's performance. After seeding cells into the microcavities, the average numbers of the cells settled at the bottom of the microcavities accounted for 72%, 97%, and 100% of the total cell numbers at 1 min, 3 min and 5 min after cells were added into the microcavities, respectively (*n* = 5), showing that the smooth sidewall surface of the microcavities facilitated the rapid cell aggregation at the microcavity bottom. **Figure** **2**a shows images of 60 3D cell spheroids formed in a microcavity array with 60 microcavities after 72 h with an average cell seeding number of 180 cells in each microcavity. The size and shape of these 3D cell spheroids were quite uniform and the cells in the spheroids exhibited tight interconnections between each other, demonstrating the good consistency between different microcavities in an array. To verify the reproducibility of different microcavity arrays in forming tumor spheroids, 110 cells were seeded into each microcavity of three different microcavity arrays with 30 microcavities in each array. The average diameters of cell spheroids formed in the three microcavity arrays after 72‐h culture were 132 ± 6 µm, 134 ± 4 µm and 128 ± 5 µm, respectively (*n* = 30) (Figure 2b; Figure S2, Supporting Information), which showed the present APHMA system had good reproducibility in forming tumor spheroids. The diameters of tumor spheroids containing an average of 110 cells in the microcavities were measured over a culture period of 7 days (n = 8) (Figure 2c). In the first 3 days after cell seeding, we observed that the cells aggregated and gradually formed compact tumor spheroids with clear appearance, and the diameters of the spheroids gradually decreased. From the fourth day, the diameters of the spheroids increased and the cells proliferated significantly. We stained the cells with calcein‐AM (live cells) and ethidium homodimer (dead cells) and continuously monitored the viability of the tumor spheroids for 7 days. The average viability of the tumor spheroids was above 88% during the 7‐day culture in APHMA (n = 5) (Figure 2d). It should be noted that this high viability monitoring result was obtained under the condition that the continuous action and accumulation of the stains on the cell spheroids had a somewhat reducing effect on the cell viability. In actual drug screening tests, tumor cell spheroids were stained only once before the viability assays, the viability of the cell spheroids could be better maintained. We also tested the status of spheroid generation with different initial cell densities by adding different average numbers of 7, 21, 56, 87, 110, and 200 of HeLa cells into different microcavities, respectively. Cell spheroids with different sizes were all formed in these microcavities with different seeding cell numbers (Figure 2e). The results of immunofluorescence staining images showed that even when the average number of cells seeded in the microcavity was 7 cells, the cells in the microcavities could form structurally intact spheroids with tight intercellular connections and intact cell–cell adhesion confirmed by the immunofluorescence image of F‐actin after 72‐h culture (Figure S3, Supporting Information). We measured the average diameters of the cell spheroids formed with representative cell numbers of 87, 110, and 180 cells were 104 ± 6 µm, 134 ± 4 µm, and 171 ± 5 µm, respectively (n = 30) (Figure 2f; Figure S4, Supporting Information). In addition to HeLa cells, APHMA could also be used to produce different types of cell spheroids including A549 lung tumor cells, HepG2 liver tumor cells, MCF‐7 breast tumor cells and HEK‐293 embryonic kidney cells. These cells could all aggregated and formed spheroid arrays after being cultured in APHMA for 72 h, indicating the broad applicability to cell types of the present system (Figure 2g). We further characterized the 3D A549 cell spheroids formed in the microcavities with average numbers of 110 cells in each microcavity using the fluorescent staining and antibody labelling method. The distribution of F‐actin and nuclei in the spheroids was also visualized by staining with phalloidin and Hoechst 33342 after 7‐day culture. The fluorescence visualization of F‐actin showed that the tumor spheroids had intact cytoskeletons and exhibited a high cellular organization and interconnection. The proliferating cells of different types of cell spheroids were labelled with the proliferation marker Ki67. The results showed that all spheroids were positive for Ki67 and the positive cells were distributed both in the core and periphery of the spheroids, indicating the presence of actively dividing cells in the entire spheroids after 7‐d culture in APHMA (Figure 2h).

In addition, we performed a comparison experiment between the APHMA system and the ultra‐low attachment (ULA) plate‐based system. Different numbers of HepG2 cells were seeded into the wells of ULA plates. Continuous imaging results showed that cells in the wells with varying seeding densities exhibited varying degrees of aggregation after 72 h, with incomplete aggregation observed in all cases. In some wells, cells remained incompletely aggregated even after 7 days (Figure S5, Supporting Information). Compared to the present microcavity arrays, which achieve 100% cell settlement within 5 min and cell aggregation within 24 h, the time required for cell aggregation to form 3D cell spheroids in the ULA plates (usually 3‐7 days) was significantly prolonged, thereby substantially delaying the time to obtain the drug sensitivity test results.

### 2.3 High‐Throughput Drug Delivery System Based on a Micropillar Array

Drug sensitivity experiments usually require the addition of different drug solutions with a wide range of concentration gradients into different test wells, which is a cumbersome, laborious and time‐consuming experimental operation. In this study, in order to expedite the process of drug sensitivity testing, we developed a micropillar array for high‐throughput drug delivery. The design of the micropillar array was illustrated in Figure S6a (Supporting Information). The micropillar array was first fabricated of polymethyl methacrylate (PMMA) sheets using the laser engraving method, with 8 micropillars fabricated on a single PMMA sheet. A complete micropillar array was assembled from 12 PMMA sheets, which had 96 micropillars, being compatible with conventional 96‐well plates. The tip of each micropillar in the micropillar arrays fabricated by the laser engraving machine had a slot structure with a slot depth of 2 mm and a width of 0.5 mm. Before drug treatments, the micropillar array was inserted into a 96‐well plate containing different drug solutions with different concentrations. The drug solutions were spontaneously introduced into the slots of the micropillar tips and quantitatively preserved in these tip slots by surface tension when the micropillar array removed from the drug solutions (Figure S6b, Supporting Information). We tested the accuracy of the micropillar array in quantitatively metering liquids using the spectrophotometry method with a detection wavelength of 509 nm and a series of carmine solutions with different concentrations as model samples. The volume of the drug solution in each micropillar was precisely controlled at 2.0 ± 0.2 µL (*n* = 8). The micropillar array loaded with different drug solutions could be immediately used for drug treatment experiments or stored for a long period of time by allowing the drug solutions loaded in the slot dry out. For drug treatment, the micropillar array loaded with different drug solutions with different concentrations was immersed in the solutions in the microcavities of APHMA containing tumor spheroids, and remained in the solutions for 30 s before being removed. The process of drug delivery could be completed within 1 min, substantially simplifying and facilitating the operation. The micropillar arrays were disposable, avoiding the potential cross‐contamination caused by reusing of the micropillar arrays.

Furthermore, we evaluate uniformity of drug solution diffusion in the hydrogel microcavities. A solution of a fluorescent dye (sodium fluorescein, 5 × 10^−4^ M) was used as a model sample to simulate the drug solution, and individually added into 10 tested microcavities. The fluorescence intensities of each well were monitored using the confocal microscopy at the Z‐stack image mode. The results showed that the fluorescein in the microcavities could reach uniform diffusion equilibrium within 5 min, which was much shorter than the drug treatment period (48 h) applied in our assays (Figure S7, Supporting Information). After 5 min, the fluorescence intensities of different microcavities tended to be uniform with a mean value of 692 ± 11 (n = 10) and an RSD of 1.5%, indicating the good consistency in distribution of the fluorescent dye among different microcavities. Considering that the molecular weight of fluorescein (376 Da) falls within a similar range as most small‐molecule drugs, it can be deduced from its diffusion behavior that small‐molecule drugs can achieve rapid and uniform dispersion within the microcavities.

### 2.4 Response of Tumor Spheroids to Different Drug Doses

Before performing the drug sensitivity assays, we validated the precision of this system in detecting drug responses in 3D spheroids, and measured the cell viabilities of A549 spheroids exposed to the 5 drug concentrations within microcavities, with five replicate tests performed at each drug concentration point. The RSDs for spheroid cell viabilities were 2.6%, 1.7%, 8.7%, 4.6%, and 4.2% at doxorubicin concentrations of 0, 0.5, 5, 10, and 25 µM, respectively (n = 5). The results demonstrated high reproducibilities of the drug sensitivity testing using the system. In addition, to further evaluate the reliability of the APHMA system in drug sensitivity testing, we performed a comparison experiment using the present system and the conventional ULA plate‐based system on drug sensitivity testing. Different concentrations of doxorubicin were treated on 3D cell spheroids in different systems. The viability of spheroids showed a similar trend between APHMA and ULA plates, with half‐maximal inhibitory concentration (IC_50_) values of 16.2 and 12.6 µM, respectively (Figure S8a, Supporting Information). Furthermore, to demonstrate the application versatility of the present system, three different types of cells were seeded into the microcavity arrays and the formed cell spheroids were treated with different concentrations of doxorubicin after 72‐h culture. The results showed different variations in the viabilities of the three types of cell samples (Figure S8b, Supporting Information), with the IC_50_ values of 29.4, 16.3, and 12.8 µM for HeLa, A549, and MCF‐7 cells, respectively.

To evaluate the drug evaluation capability of the APHMA platform, a 5‐day drug sensitivity testing was performed. HeLa spheroids were tested for drug sensitivity to three anticancer drugs with different therapeutic mechanisms, including doxorubicin, sorafenib, and cisplatin. Doxorubicin inhibits RNA and DNA synthesis; sorafenib inhibits multiple kinase activities affecting cell signaling pathways; cisplatin inhibits cell mitosis by disrupting DNA function through binding to DNA. Tumor cells were continuously grown in APHMA for 72 h to form spheroids before drug treatments. The tumor spheroids were treated with different drug solutions for 48 h, using the micropillar array for drug delivery. Three replicates were performed for each drug concentration, and negative control (DMSO) and positive control (benzalkonium chloride) were included in the same batch of screening.

**Figure 3:**
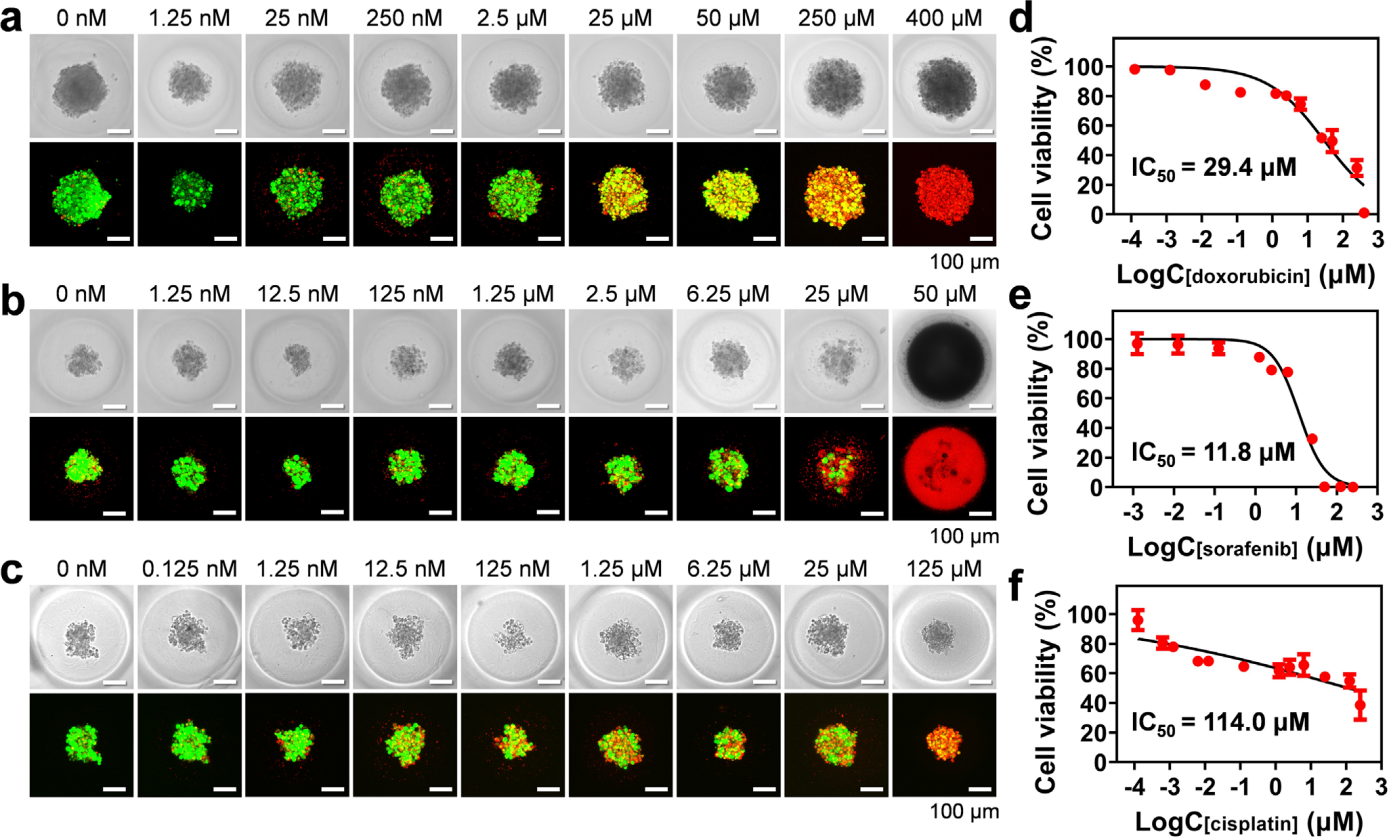
Mono‐drug treatments in APHMA. a–c) Fluorescence images of the HeLa spheroids stained with calcein‐AM (green, live cells) and ethidium homodimer (red, dead cells) after 48 h of different doses of doxorubicin (a), sorafenib (b) and cisplatin (c). Scale bars, 100 µm. d–f) Variations of the spheroid viability with the concentrations of doxorubicin (d), sorafenib (e) and cisplatin (f) (n = 3). Three replicates were performed for each drug concentration.

The HeLa tumor spheroids were stimulated with different concentration ranges up to 6 orders of magnitude of doxorubicin, sorafenib, and cisplatin, respectively. The fluorescence images of cell spheroids under the treatments of different drugs are shown in **Figure** **3**a–c. The cell viability showed significant changes with the drug concentrations. The IC_50_ values for doxorubicin, sorafenib, and cisplatin were determined^[^ ^34^ ^]^ as 29.4, 11.8, and 114.0 µM, respectively (Figure 3d –f). These results are consistent with those reported previously.^[^ ^35^, ^36^, ^37^ ^]^

**Figure 4:**
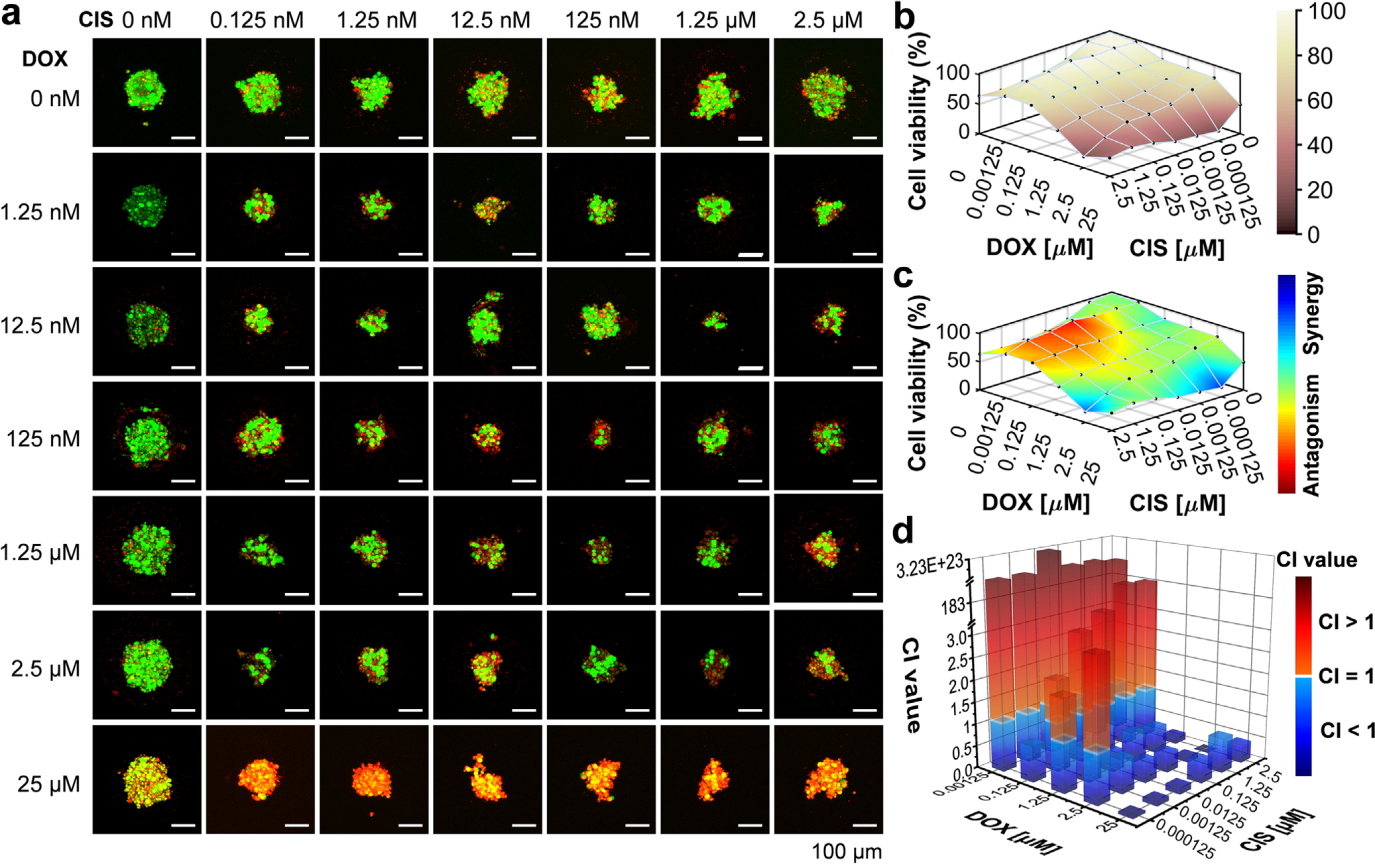
Tests of drug combination of doxorubicin + cisplatin for HeLa spheroids in APHMA. a) Fluorescence images of HeLa spheroids stained with live (green) cells and dead (dead) cells stimulated with the doxorubicin + cisplatin combination for 48 h. Scale bars, 100 µm. b) Viability of the spheroids after treatments with the doxorubicin + cisplatin combination. c) The therapeutic efficacy of doxorubicin + cisplatin at different doses were analyzed by Bliss model. d) CI values of HeLa spheroids with doxorubicin and cisplatin at different doses determined by the CompuSyn software. Data shown as mean ± SD for all spheroids.

**Figure 5:**
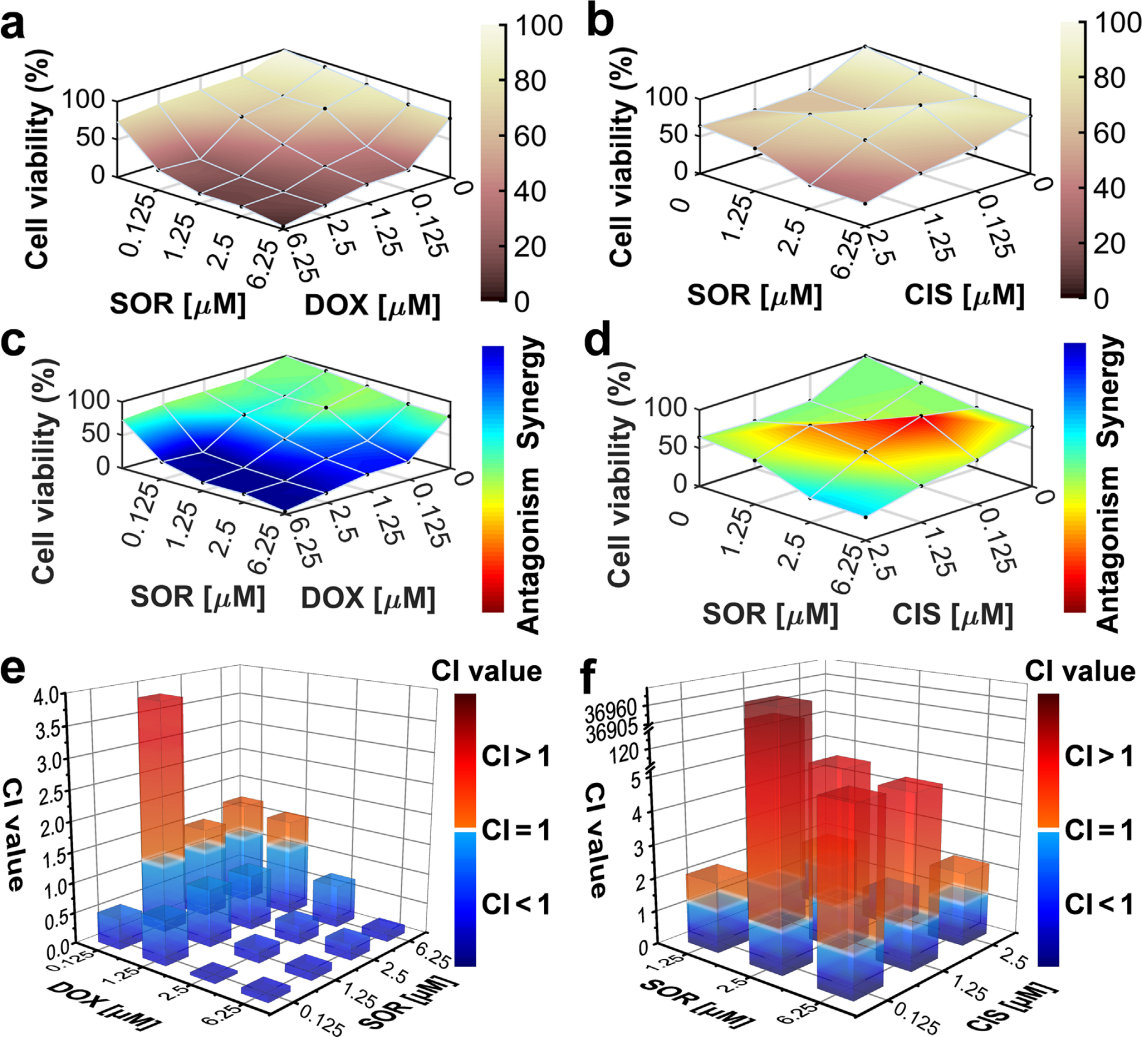
Tests of drug combinations of sorafenib + doxorubicin and sorafenib + cisplatin for HeLa spheroids in APHMA. a,b) Viability of the spheroids after treatments with drug combinations of sorafenib + doxorubicin (a) and sorafenib + cisplatin (b). c,d) The therapeutic efficacy of sorafenib + doxorubicin (c) and sorafenib + cisplatin (d) at different doses were analyzed by Bliss model. e,f) CI values of HeLa spheroids with sorafenib + doxorubicin (e) and sorafenib + cisplatin (f) at different doses determined by the CompuSyn software. Data shown as mean ± SD for all spheroids.

Compared to mono‐drug treatment, drug combination aims to inhibit multiple redundant pathways of tumor cells and has been widely accepted for better efficacy.^[^ ^38^ ^]^ Determination of drug combinations depending on screening is an indispensable procedure for effective or even optimal treatment.^[^ ^39^ ^]^ To test the feasibility of the present system in high‐throughput drug combination screening, we conducted drug stimulation on HeLa spheroids with the pairwise combinations of three drugs of doxorubicin, sorafenib, and cisplatin. Compared to the spheroids without drug treatments, the spheroids after drug stimulation exhibited lower activity in most concentration combinations (**Figures** **4**a,b and **5**a,b). The effects of different drug combinations were analyzed using the Bliss model. The combination of doxorubicin and sorafenib showed stronger synergistic effects at most concentration combinations. However, the synergistic effects of doxorubicin and cisplatin, as well as sorafenib and cisplatin, were not significant (Figures 4c and 5c,d). We further quantified the synergism effect of the three combinations by calculating the combination index (CI) using the CompuSyn software,^[^ ^40^ ^]^ with the CI values less than 1, equal to 1, and greater than 1 indicating synergism, additive, and antagonism effect, respectively. The results showed that the CI values for most combinations at the doxorubicin concentrations between 1.25 and 25 µM were less than 1, indicating there was a synergistic effect between doxorubicin and cisplatin on the HeLa spheroids (Figure 4d; Table S1, Supporting Information). In addition, there was strong synergism between doxorubicin and sorafenib, but antagonism effect between sorafenib and cisplatin (Figure 5e,f; Tables S2 and S3, Supporting Information). These results are also consistent with those previously reported.^[^ ^41^, ^42^ ^]^

### 2.5 Patient‐Derived Breast Primary Cells Drug Screening in APHMA

Breast cancer is the most common cause of cancer death among women worldwide. There are more than 20 different subtypes of breast cancer, which are genetically, morphologically, and clinically distinct. Currently, the treatments of breast cancer are mainly based on clinical pathological features. Due to the heterogeneity and complexity of breast cancer, the widely‐used treatments based on pathological features are difficult to be tailored to individual patients, reducing the effectiveness of treatment. The vitro personalized drug testing on the cell samples of individual patients provides an effective strategy to accurately predict clinical outcomes for patients. In this study, we performed rapid, high‐throughput culture and drug testing for patient‐derived breast cancer primary cells in APHMA.

**Figure 6:**
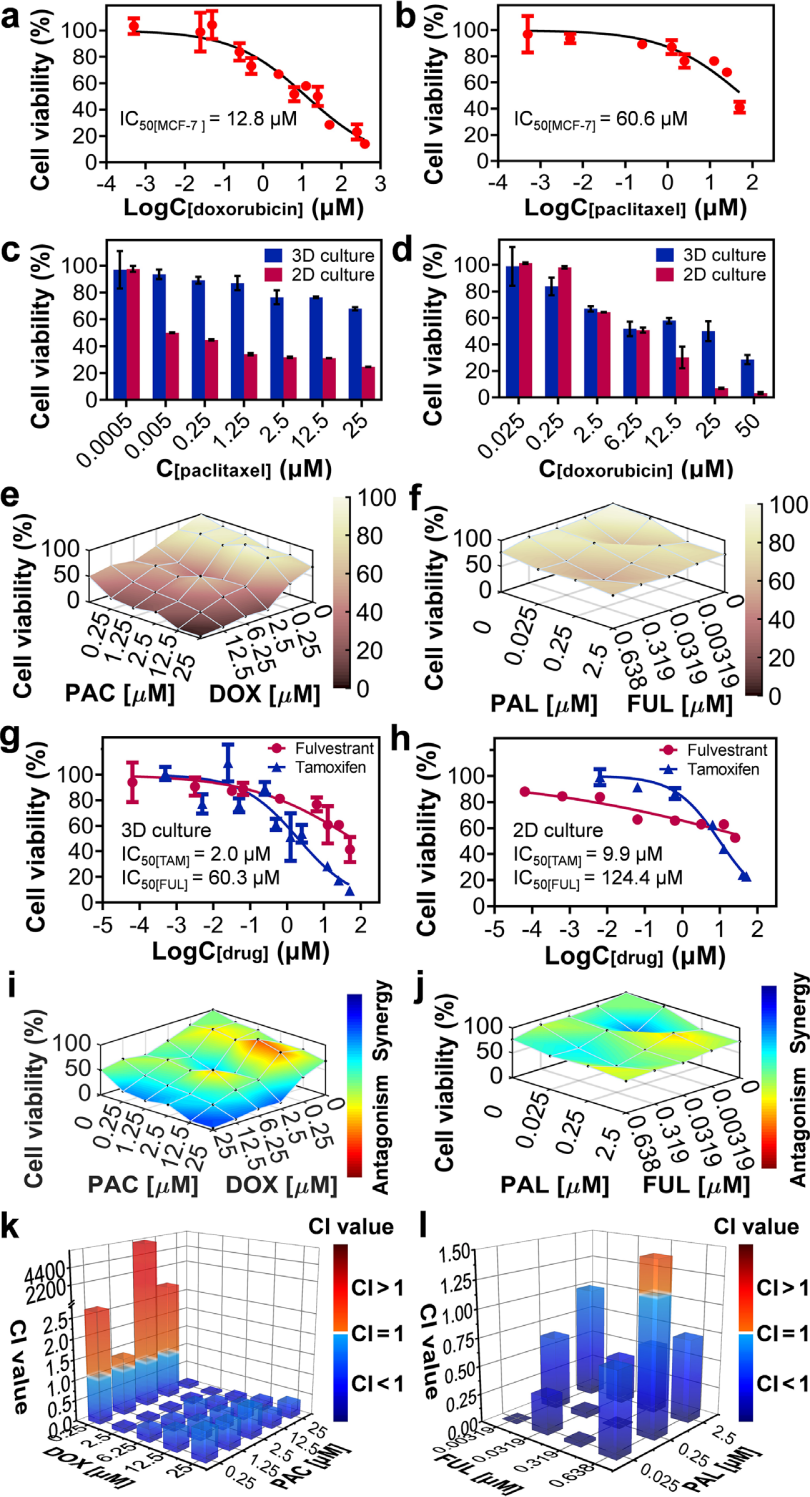
Drug treatments of MCF‐7 spheroids in APHMA. a,b) Cell viability of the MCF‐7 spheroids at different doses of doxorubicin (a) and paclitaxel (b). c,d) Comparison of 2D‐ and 3D‐cultured MCF‐7 cells stimulated by paclitaxel (c) and doxorubicin (d). e,f) Viability of the MCF‐7 spheroids stimulated by the combinations of doxorubicin + paclitaxel (e) and fulvestrant + palbociclib (f). g) Cell viability of the MCF‐7 spheroids at different doses of fulvestrant and tamoxifen. h) Cell viability of 2D‐cultured MCF‐7 cells at different doses of fulvestrant and tamoxifen. i,j) Therapeutic efficacy of doxorubicin + paclitaxel (i) and fulvestrant + palboliclib (j) at different doses analyzed by the Bliss model. k,l) CI values of the MCF‐7 spheroids with doxorubicin + paclitaxel (k) and fulvestrant + palbociclib (l) at different doses calculated by the CompuSyn software. Data shown as mean ± SD for all cells.

Prior to drug treatments to clinical patient samples, we first performed drug sensitivity testing with breast cancer cell lines. The estrogen receptor‐positive (ER+) breast cancer is the most common breast cancer subtype,^[^ ^43^ ^]^ accounting for more than 70% of all breast cancer cases.^[^ ^44^ ^]^ Patients with this type usually receive endocrine therapy and chemotherapy.^[^ ^45^ ^]^ Therefore, we first carried out the drug sensitivity testing on the MCF‐7 breast tumor spheroids with estrogen receptor‐positive expression using two commonly‐used chemotherapy drugs of doxorubicin and paclitaxel. Paclitaxel inhibits cell growth by stabilizing and enhancing the polymerization of tubulin, preventing microtubule depolymerization and inhibiting cell mitosis. After 72 h of culture, the tumor spheroids were stimulated with doxorubicin and paclitaxel solutions with different concentrations for 48 h, with both negative and positive control groups. The IC_50_ value of doxorubicin against MCF‐7 tumor spheroids was 12.8 µM and the IC_50_ value of paclitaxel was 60.6 µM (**Figure** **6**a). As previously reported in literature,^[^ ^46^ ^]^ the IC_50_ values for doxorubicin for MCF‐7 spheroids in 3D culture were in the range of 0.2–50 µM under different experimental conditions. We further compared the above results in APHMA with those obtained with 2D‐cultured cells in traditional 96‐well plates. At most concentrations of both paclitaxel and doxorubicin, the cell viability of 3D‐cultured cell spheroids was higher than that of 2D‐cultured cells, with the difference being more apparent in the paclitaxel experiments (Figure 6c,d). These results are consistent with those previously reported,^[^ ^47^ ^]^ and demonstrated that 3D‐cultured tumor spheroids, compared to 2D‐cultured cells, had enhanced drug resistance and thus could better simulate the in vivo response to drug stimulation.

Endocrine therapy has become one of the standard treatments for most ER+ breast cancer patients because it can improve patients’ survival rate and quality of life.^[^ ^48^ ^]^ Tamoxifen and fulvestrant are both estrogen receptor antagonists, which are frequently used in breast cancer treatments. They are structurally similar to estradiol and can compete with estradiol for binding to the estrogen receptor, thereby inhibiting tumor cell growth. In addition, fulvestrant can also accelerate the degradation of estrogen receptor proteins, downregulate the expression of estrogen receptors, and inhibit the estrogen receptor signaling pathway. For endocrine drug sensitivity tests, the cultured MCF‐7 tumor spheroids were respectively stimulated with multiple doses of tamoxifen and fulvestrant. The IC_50_ values of tamoxifen and fulvestrant on MCF‐7 spheroids were 2.0 and 60.3 µM, respectively (Figure 6g). Similarly, as a comparison, 2D culture and endocrine drug sensitivity tests of the same batch of MCF‐7 cells were performed, with the IC_50_ values for tamoxifen and fulvestrant of 9.9 and 124.4 µM (Figure 6h). Tamoxifen and fulvestrant act by binding to estrogen receptors and interfering with estrogen signaling pathways, i.e., their efficacy depends not only on drug concentrations but also on the endogenous receptor expression levels within the sample cells.^[^ ^49^, ^50^ ^]^ This may lead to inconsistencies between the results of their comparative experiments using 3D and 2D cultured cells and the experimental results of chemotherapy drugs.

Based on the results of mono‐drug testing, we further employed the drug combination method to perform the drug sensitivity testing for MCF‐7 breast tumor spheroids, which has been shown to be able to improve the therapeutic efficacy and reduce tumor resistance and is being used more and more widely in clinical treatment. Doxorubicin and paclitaxel were selected as the chemotherapy drugs, while fulvestrant and palbociclib were chosen as the endocrine therapy drugs to stimulate spheroids in APHMA. Palbociclib is a new drug for the treatment of breast cancer with action mechanism of inhibiting the growth and division of breast cancer cells by inhibiting cyclin‐dependent kinases 4 and 6 (CDK‐4/6). In the experiments with the combinations of doxorubicin and paclitaxel, the combined drugs at different doses showed stronger killing effect to tumor cells compared to the mono‐drug experiments (Figure 6e). Through the Bliss model analysis, we evaluated there was a synergistic effect under the two drug combinations (Figure 6i). Furthermore, the results of CI values analyzed by CompuSyn indicated that 84% of CI values among all concentration combinations were less than 1, indicating the obvious synergistic effect between doxorubicin and paclitaxel, which were consistent with previous reports^[^ ^51^ ^]^ (Figure 6k; Table S4, Supporting Information). Among them, the combination of 2.5 µM doxorubicin and 25 µM paclitaxel showed the strongest synergism effect. In the combination drug tests of 2D‐cultured cells, the results were different, with doxorubicin + paclitaxel exhibiting synergism effect at low concentrations, but antagonism effect at high concentrations (Figure S9a–c; Table S6, Supporting Information). This might be caused by the differences in the cell growth microenvironment under 2D and 3D culture conditions, which led to differential responses to the same drug. The endocrine combination of fulvestrant and palbociclib showed more effective than the mono‐drug tests, as previously reported^[^ ^52^ ^]^ (Figure 6f). The synergistic effect was strongest when the concentration of fulvestrant was 3.19 nM and the concentration of palbociclib was 25 nM among all 12 combinations (Figure 6j). Similarly, the results of the CI values confirmed the results of the Bliss model (Figure 6l; Table S5, Supporting Information). In endocrine combination drug tests of 2D‐cultured cells, synergism effects were observed in drug combinations with high concentrations of fulvestrant (Figure S9d–f; Table S7, Supporting Information).

**Figure 7:**
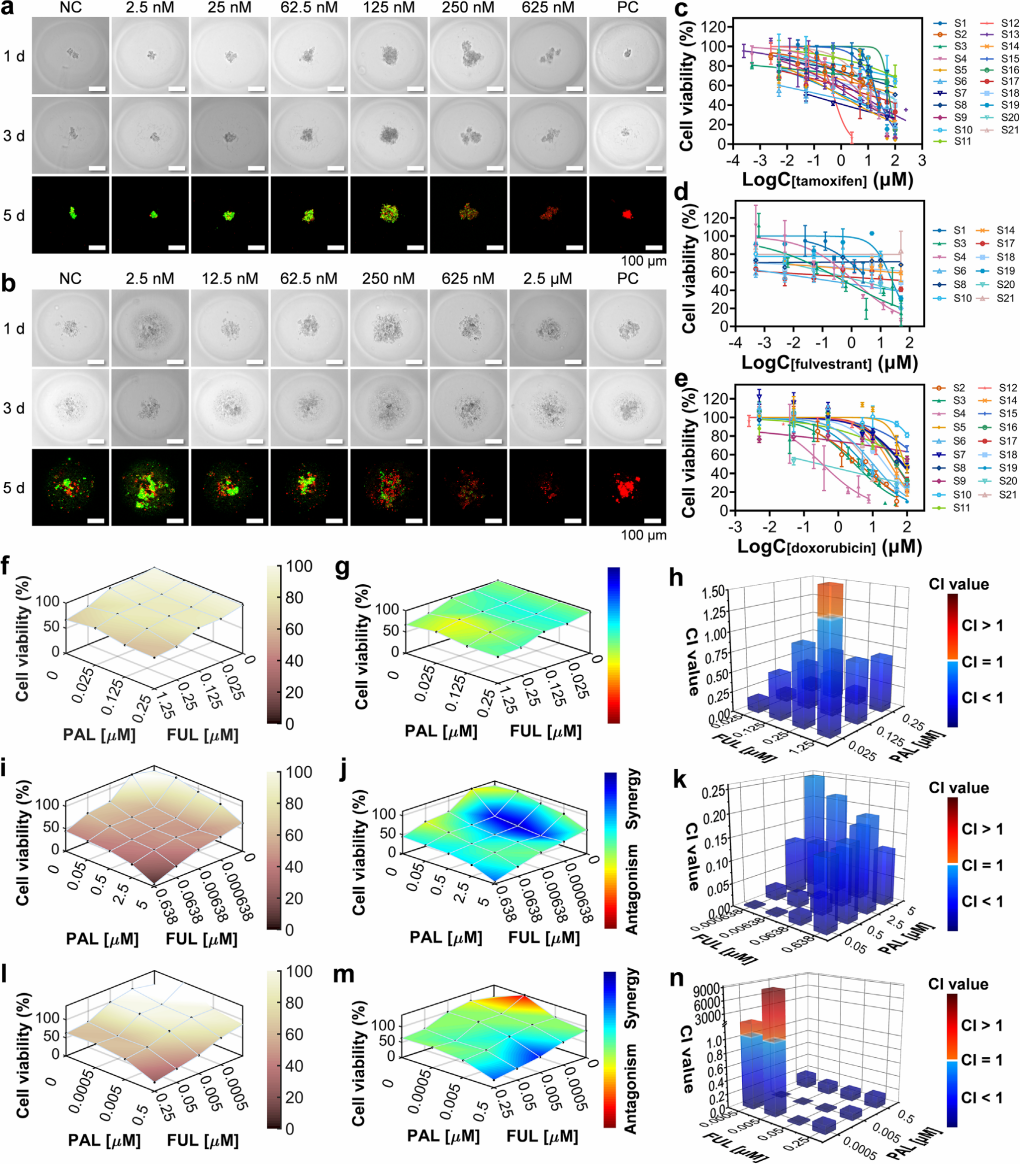
Drug sensitivity testing for patient‐derived breast primary cell samples. a,b) Bright field images and live/dead staining images of cell spheroids in microcavities with the samples of patient S12 with cell size ranges of 37–100 µm (a) and <37 µm (b) under different doses of tamoxifen stimulation at 1st day, 3rd day, 5th day. NC: negative control (DMSO), PC: Positive control (benzalkonium chloride). Scale bars, 100 µm. c) Cell viabilities of the samples (cell size <37 µm) from 21 patients stimulated with different doses of tamoxifen for 72 h. d) Cell viabilities of the samples from 12 patients with different doses of fulvestrant for 72 h. e) Cell viabilities of the samples from 19 patients stimulated with different doses of doxorubicin for 72 h. f,i,l) Cell viability of the samples from patients S1 (f), S3 (i) and S4 (l) with drug combination of fulvestrant + palbociclib. g, j, m) Treatment efficacy to the samples of patients S1 (g), S3 (j) and S4 (m) with fulvestrant + palbociclib at different doses analyzed by the Bliss model. h, k, n) CI values of the samples of patients S1 (h), S3 (k), S4 (n) with fulvestrant + palbociclib at different doses. Data are shown as mean ± SD for all spheroids.

Based on the above approach and results, we carried out rapid, high‐throughput 3D cell culture and drug sensitivity testing of patient‐derived tumor cells from 21 clinical patients using the APHMA system. The detailed clinical information on each patient is provided in Table S8 (Supporting Information). According to the clinical histological diagnosis, patients S1, S3, S4, S5, S6, S8, S9, S10, S11 had invasive ductal carcinoma (IDC), patient S2 and S7 had ductal carcinoma in situ (DCIS) of the breast, and patients S12–S21 had breast fibroadenoma (FA). Specifically, patients S1 and S3 were triple‐negative breast cancer patients and were negative for estrogen receptor (ER), progesterone receptor (PR), and human epidermal growth factor receptor 2 (HER2). Patients S5 and S9 were positive for ER, PR and HER2. Patient S2 and S8 was ER and PR negative and HER2 positive. Patients S12–S21 were positive for ER. Patient S4 was ER and PR positive and HER2 negative. In the clinical management, these pathologically informed results determine the patient's medication regimen. First, to verify whether the patient‐derived primary cells could be cultured and performed drug treatments in APHMA, we selected two different cell size ranges, smaller than 37 µm and 37‐100 µm, for the preliminary drug testing, among which the former consisted mainly of single cells, the latter mainly of small clusters of cells. The surgically excised tissues from the patients were sequentially processed to obtain the two types of cell samples. The bright field images and live/dead staining images of two different sizes of cells under different doses of tamoxifen stimulation from patient S12 in the microcavities at 1st day, 3rd day, 5th day after seeding are shown in **Figure** **7**a,b. Both sizes of primary cells fully aggregated at the bottom of the microcavities within 1 day. However, the spheroids of the patient‐derived tumor cells were less tightly clustered compared to the cancer cell lines. This may be because the primary cells from the breast cancer patients contained other types of cells in addition to the tumor cells, such as some fibroblasts. The live/dead staining results showed that the cell viability of both sizes of cells after 5 days in the microcavity was 84% and 76%, respectively, and the cell viability decreased as the doses of tamoxifen increased. It is worth noting that for experiments using primary cells derived from patients, we increased the number of cells within each microcavity to 200–300 cells to ensure the consistency in 3D cellular structure and intercellular interactions across different microcavities while preserving the microenvironment of the primary tumor cells where possible. These cell seeding numbers were determined by balancing the availability of the patient‐derived samples with the need to complete drug screening experiments under both mono‐drug and drug combination treatment conditions. To evaluate the efficacy of primary cell 3D‐culture within the microcavities, we performed immunofluorescence staining on the patient‐derived primary cells cultured for one week in the microcavities. The results demonstrated the formation of distinct and widespread intercellular connections among the patient‐derived primary cells (Figure S10, Supporting Information).

We conducted the drug sensitivity testing of mono‐drug and drug combination treatments to the clinical samples. Both chemotherapy and endocrine therapy were performed on different patients. Generally, clinical dosing regimens are formulated based on patients’ pathological information. However, in order to shorten the clinical waiting time, drug tests in vitro were performed immediately after surgery and completed within 5 days in this work, which was earlier than the time when the clinical pathology results were available. Therefore, we tested both the chemotherapy and endocrine drug regimens on the primary cell samples. The primary cell samples less than 37 µm from the 21 patients were stimulated with different doses of tamoxifen (Figure 7c). The IC_50_ values of tamoxifen for samples of patients S4, S5, S7, S9 and most breast fibroadenoma were slightly lower than those of patients S1, S2, S3, and S8, suggesting that tamoxifen had a stronger inhibitory effect on cellular activity of the former samples. In general, tamoxifen is more effective in treating estrogen receptor‐positive patients than estrogen receptor‐negative patients. This further demonstrated that our results were in line with the clinical findings. Patient S4, an ER‐positive patient, has been treated with endocrine therapy with tamoxifen in the postoperative period, with no tendency to recurrence within 4 courses of treatment. We also performed drug treatments with different doses of fulvestrant on 12 patients (Figure 7d). Fulvestrant was more effective for samples of patient S1, S3, S4, S6 and S20 compared to other patients under treatments. This result implied that it might have the possibility for these 5 patients to try fulvestrant for better therapeutic outcome. Since neoadjuvant chemotherapy has become one of the common postoperative treatments for clinical breast cancer patients, we also performed doxorubicin stimulation to the samples of 19 patients. Doxorubicin was more effective in inhibiting cellular activity in samples of patients S2, S3, S4, S19, S20 and S21 compared to other patients (Figure 7e). In the postoperative period, patient S3 has been treated with doxorubicin‐based chemotherapy and did not experience a recurrence of anthracycline therapy within 4 sessions, which was consistent with the results of our drug testing. The IC_50_ values of all patients with mono‐drug treatments at different doses are shown in Table S9 (Supporting Information). In addition, for patients S1, S3, and S4, who still had samples available after the mono‐drug testing, we also tested their samples with the drug combination of fulvestrant and palbociclib. The testing results are as shown in Figure 7f–n. Compared to mono‐drug treatment of fulvestrant, the combination of fulvestrant and palbociclib showed strong synergistic effects at some combination concentrations for patients S3 and S4. This suggested that the choice of clinical dosing regimen for these two patients should perhaps consider the concentration matching of the two co‐administered drugs. For patient S1, the drug combination showed additive effects or weak synergistic effects at most concentrations, thus the combination of fulvestrant and palbociclib was not recommended in clinical drug treatment. These results were also consistent with the CI values for each combination (Figure 7h,k,n; Tables S10–S12, Supporting Information).

## 3 Discussion

In this work, we developed the air‐punched fabrication approach for nanoliter‐scale microcavity arrays with ultra‐smooth microcavity surfaces, with which rapid formation and 3D culture of tumor cell spheroids from very small numbers of cell samples, as well as the subsequent high‐throughput drug sensitivity testing could be achieved within 5 days. The non‐contact airflow impacting approach used flowing air as a fluid mold to rapidly generate microcavities with ultra‐smooth inner surfaces, unlike other previously‐reported microcavity fabrication techniques that rely on solid‐state molds,^[^ ^23^, ^53^ ^]^ and thus avoided defects or residues associated with the solid‐state molds in contact with the gel and during demolding. The nanoliter‐scale microcavities with smooth surfaces significantly facilitated the physical aggregation of cells in seconds and rapid cell spheroid formation and culture in the microcavities, enabling minimal sample cell numbers as low as 10 cells per microcavity, rapidest 3D spheroid‐forming culture in 24 h, and high‐throughput and large‐scale drug sensitivity testing as fast as 5 days. The system had a wide range of cell sample adaptability, different types of tumor cell lines and primary tumor cell samples from clinical patients could be used to generate spheroid arrays for drug sensitivity testing. These features make the present system ideally suited for very rapid drug sensitivity testing directly using limited numbers of rare primary clinical samples without the need for prolonged‐term culture and proliferation of primary cells, and have the potential to provide useful information for individualized precision medication.

In addition, we applied the system in rapid, high‐throughput drug sensitivity testing on primary cell samples from 21 clinical breast cancer patients to quantify the responses of patient‐derived cells to chemotherapy drugs and endocrine drugs under both the mono‐drug and combinational drug treatment modes. The observed consistency between the APHMA‐derived drug sensitivity results and clinical pathology observations offers preliminary evidence supporting the potential translational relevance of the system. Compared to conventional 2D and 3D drug sensitivity testing and screening systems, the volume of cell suspension consumed in each microcavity of this system was only 300 nL, and the numbers of cells used for drug treatment in each microcavity were only tens to hundreds of cells, which are approximately 20 times lower than the conventional multiwell plate systems.^[^^20,21]^ This makes it possible to use biopsy samples with small amounts of cell (e.g., tens of thousands of cells) to perform high‐throughput and large‐scale drug sensitivity screening without the need for pre‐expansion of the cell samples. Compared to those expansion culture systems including the organoid‐based systems, our system does not require tens of days of prolonged culture and extensive proliferation of primary cells, and enables rapid spheroid‐forming 3D culture of primary cells in a much shorter time period of 72 h. With the present platform, the entire workflow from clinical sample collection, cell seeding, 3D spheroid‐forming culture, stimulation with different types, concentrations and combinations of drugs, to drug sensitivity result readout could be completed in only 5 days, which is the shortest drug sensitivity testing time for 3D culture cells reported in the literature to date.^[^ ^54^, ^55^ ^]^ For a single patient biopsy sample with only ≈40000 primary cells, 144 tests could be performed for screening up to 4 single drugs and 2 drug combinations, where each drug condition was tested at 10 drug concentrations spanning 6 orders of magnitude. The features of less sample amounts, shorter testing time, and easily‐achievable high‐throughput and large‐scale screening, making it ideal for very rapid drug sensitization experiments directly using a limited number of rare primary clinical samples, and has the potential to provide useful inspiration and guidance for clinical precision medicine treatment.

In addition to tumor cells, the system can be used to culture and test other cell types, as well as serve as a platform for organoids or organs‐on‐a‐chip experiments for a wider range of applications, such as large‐scale high‐throughput drug screening for drug discovery or fundamental biomedical research, in which the consumption and cost of the reagents and samples can be dramatically reduced.

While the present study demonstrates the strong technical potential of the APHMA platform for rapid, low‐sample, high‐throughput drug sensitivity testing, we acknowledge that this work represents an early‐stage technological development. Although encouraging concordance was observed between the APHMA‐derived responses and clinical pathology, the current patient cohort remains limited in scale, and further validation using larger, multi‐type clinical datasets will be essential to establish predictive accuracy and clinical robustness. Additional work will also be required to standardize procedures for sample handling, analytical performance assessment, and assay reproducibility under clinically regulated conditions. Furthermore, clinical risk assessment will be necessary to realize the translational potential of the APHMA system.

## 4 Experimental Section

### Construction of the APHMA Platform

The APHMA platform consisted of four parts: an air pressure source module, an air flow transmission module, a microcavity array formation module and a system control module. The air pressure source module mainly consisted of an air compressor (ED‐0204, EIDOLON, Jinhua, China) to provide positive pressure air flow. The air flow transmission module included four sections: polyurethane tubes (8 mm o.d., 5 mm i.d., JEND, Wuxi, China) for transmitting positive air flow, a gas pressure regulating valve (IR1000‐01, Splan, Wenzhou, China) for adjusting the airflow rate to a suitable level, an air filter (S593CSFTR‐0.2H83SH83SN8, CAPSID, Dalian, China) for filtering and sterilizing the air flow, a stainless steel injection needle (510 µm o.d., 260 µm i.d., Desai, Jiaxing, China) used as the outlet of the air flow. The stainless‐steel needle with a flat tip was fixed on an automated z‐translation stage (SKR series, THK, Tokyo, Japan). The microcavity array formation module consisted of a 96‐well plate fixed on an automated x‐y translation stage (SKR series, THK, Tokyo, Japan), in which the hydrogel microcavity array was generated. The system control module was used to control the operations of the other modules in the platform.

### Generation of APHMA

The hydrogel microcavity array was formed using the air punching approach. Briefly, a 1.5% (w/v) agarose hydrogel solution (Biowest) was sterilized and kept in a water bath at 40 °C to maintain it in the liquid state. Before the generation of the hydrogel microcavity array, 100 µL of the agarose hydrogel solution was added to each well of a 96‐well plate (3599, Corning Co., New York, USA) using a pipette. The microcavity hydrogel in each well was formed by first moving the translation stages to allow the tip of the stainless‐steel needle align with the center of the well, with the tip a set distance from the upper surface of the hydrogel gel solution inside the well. Then, the air compressor was switched on and a stable airflow with a set flow rate and a temperature of 25 °C which was lower than that of the gel solution was ejected from the needle tip, continuously impacting on the gel solution for 30 s, to form a solidified nanoliter‐scale microcavity in the gel solution. By repeating the above operation for different wells in the 96‐well plate, a hydrogel microcavity array could be fabricated.

### Cells and Cell Culture

MCF‐7 (ATCC HTB‐22), HeLa (ATCC CCL‐2), A549 (ATCC CCL‐185), HepG2 (ATCC HB‐8065), and HEK‐293 (ATCC CRL‐1573) were obtained from the Cell Bank of the Chinese Academy of Sciences (Shanghai, China). All cell lines were maintained in T25 culture flasks (430639, Corning Co., New York, USA) with 8 mL of Dulbecco's Modified Eagle Medium (DMEM, GENOM) supplemented with 10% (v/v) fetal bovine serum (FBS, VivaCell), 1% (v/v) penicillin/streptomycin (Gibco). The flasks were incubated in the incubator at 37 °C with a constant 5% CO_2_ supply. When cells reached nearly 90‐95% confluence, new passages were performed every 3‐4 days. To passage the cells, the cells were rinsed with phosphate buffered saline (PBS, Gibco) and detached from the flasks using 1 mL 0.25% trypsin‐EDTA (GENOM) solution for 2 min. All cells were uncontaminated and they were used for experiments prior to passage 10.

### Human Breast Cancer Tissue and Fibroadenoma Collection

Human cancer tissue and fibroadenoma, confirmed by a pathologist, were collected from the Second Affiliated Hospital of Zhejiang University. The collection of removed tissues was approved by the ethics review committee of the Second Affiliated Hospital of Zhejiang University School of Medicine (I2021001303, IR2021001330), and informed consent for collection and research was obtained from each patient before surgery. The informed consent was obtained from the patients and the clinical characteristics of each patients are available in Table S8 (Supporting Information). Breast tissues stored in a preservation solution (Advanced Dulbecco's modified Eagle's medium/Nutrient Mixture F12 (Advanced DMEM/F12, Gibco) containing 1% penicillin and streptomycin) were transported to the laboratory and processed within 1 day of removal from the patients. Tissues were cut into small pieces with surgical scissors and resuspend the tissue pieces in DMEM/F12 in gentleMACS c‐tubes (130‐096‐334, Miltenyi Biotec Co., Bergisch Gladbach, Germany). Samples were dissociated by a gentleMACS octo Dissociator with Heaters (130‐096‐427, Miltenyi Biotec Co., Bergisch Gladbach, Germany) for 1 h. The obtained sample was centrifuged and resuspended in advanced DMEM/F12 containing 1% penicillin and streptomycin. The digested tissue suspension was strained over a 100 µm filter (27 217, STEMCELL Technologies Inc., Vancouver, Canada) and pelleted at 400×g for 10 min. Then, 2 mL red blood cell lysis buffer (BOSTER) was added and erythrocytes were lysed for 5 min at room temperature. The suspension was filtered through a 100‐µm and a 37‐µm filter (27215, STEMCELL Technologies Inc., Vancouver, Canada), respectively. The cell suspension passing through the 37‐µm filter and the cell clusters in the range of 37–100 µm were collected, respectively.

### Cell Seeding

Cell suspension was obtained after trypsinization, centrifugation, and resuspension processes. The cell density was calculated using a hemocytometer. For 2D cells seeding, 100 µL of cell suspension (3.0 × 10^4^ cells mL^−1^) was loaded into each well of the 96‐well plate and cultured in an incubator at 37 °C with a constant 5% CO_2_. For 3D cells seeding, the operation was completed based on the SODA system built in the authors’ group.^[^ ^31^, ^32^ ^]^ Specifically, in the experiments, the cell suspension with specific cell densities was first loaded into a well in a 48‐well plate as the sample well. During the entire process of cell seeding, the cell suspension in the sample well was continuously stirred magnetically to prevent cell sedimentation. For the cell seeding operation, 1400 nL cell suspension in the sample well was aspirated into the capillary probe (250 µm o.d., 150 µm i.d., Refined Chromatography Co., Yongnian, Handan, China) connected with a homemade syringe pump with a 10 µL syringe (1701 N, Hamilton, Reno, USA) in the SODA system. A portion of 100 nL of the cell suspension in the capillary probe was immediately dispensed into the waste well containing PBS solution. Subsequently, four 300 nL cell suspension in the capillary probe were sequentially added to four individual microcavities. Finally, the remained ≈100 nL of cell suspension in the capillary probe was completely dispensed into the waste well. The above operations for cell seeding of four microcavities could be completed within ≈10 s. Within such a short timeframe, cells aspirated into the capillary probe would not undergo significant sedimentation, thereby ensuring consistency in the number of cells added to each well. The above operation was repeated to complete cell seeding in the other microcavities of the microcavity array. The microcavity array seeded with cell samples was placed in an incubator at 37 °C with 5% CO_2_ for compact tumor spheroids formation.

### Fabrication of Micropillar Array

The micropillar arrays were used for achieving simple and efficient drug delivery. The micropillar arrays were made from PMMA plates with a thickness of 2 mm by a laser engraving machine (CMA6040, GD Han's Yueming Laser Group Co., Dongguan, China). First, the configuration of the micropillars was designed with AutoCAD (Autodesk Co., SAN Rafael, USA). On one PMMA plate, 8 micropillars were fabricated using the laser engraving machine according to the configuration design, with a width of 2 mm and a length of 14 mm for each micropillar, and a slot of 0.5 × 2 mm was fabricated at the tip of each micropillar. A 12 × 8 channel micropillar array was produced by assembling 12 PMMA plate with 8 micropillars on each plate, with the aid of a homemade 96‐hole plate for matching the traditional 96‐well plate. Before drug tests, the micropillars of the micropillar array were washed three times with sterile water for 5 min, soaked in 75% ethanol for 5 min for three times and stored in a sterile environment. When performing drug sensitivity testing, the micropillar arrays were inserted into a 96‐well plate containing different drug solutions, and then removed from these solutions. The drug solutions were spontaneously introduced into the slots of the micropillars by surface tension. High‐throughput drug delivery to cell samples was realized by inserting the micropillars loaded with the drug solutions into the corresponding microcavities or wells where cells were cultured.

### Drug Treatments

Mono‐drug treatments and drug combination experiments were performed under the 2D and 3D mode using seven clinically relevant chemotherapy and endocrine therapy compounds: doxorubicin HCl (Selleckchem), sorafenib (Solarbio), cisplatin (Solarbio), paclitaxel (Selleckchem), tamoxifen (Selleckchem), fulvestrant (Selleckchem), palbociclib HCl (Selleckchem). All of these compounds were dissolved in DMSO (Solarbio) and delivered at set final concentrations in culture medium with a maximal final DMSO concentration of 0.1% v/v or less.

For chemotherapy, cells were cultured on 96‐well plates under the 2D mode and on APHMA and ULA plates (7007, Corning Co., New York, USA) under the 3D mode for 72 h, respectively, followed by different doses of drug treatments for a further 48 h. For endocrine therapy, MCF‐7 cells were starved in stripped medium for 24 h. The stripped medium was phenol‐red free (PRF) DMEM (Gibco) medium supplemented with 5% charcoal‐stripped serum (CSS, VivaCell), 1% glutamax (Gibco), 1% non‐essential amino acid (Gibco), 1% essential amino acid (Gibco), 1% sodium pyruvate (Gibco), and 1% penicillin‐streptomycin. The MCF‐7 cells were digested with phenol‐red free trypsin (VivaCell) to obtain cells in stripped medium. Cells were grown in APHMA or 96‐well plates for 72 h to form spheroids or 2D adhesive cells in the stripped medium, followed for a further 48 h drug treatments.

### Measurement of Viability of Tumor Spheroids in APHMA

The viability of tumor spheroids in the APHMA was measured using the live/dead staining method with the LIVE/DEAD Viability/Cytotoxicity Kit (Invitrogen). The staining solution was prepared by adding 5 µL calcein AM and 20 µL ethidium homodimer‐1 to 10 mL DPBS (Gibco). The measurement was conducted by first aspirating the media out from the microcavity and replacing it with 50 µL staining solution. After 1 h of incubation at 25 °C, the cell spheroids were imaged using a confocal microscope (A1R HD25, Nikon Co., Tokyo, Japan).

### 4.1 Image Analysis

The confocal z‐stack images of the cell spheroids were analyzed with NIS‐Elements AR 5.01 software (Nikon Co., Tokyo, Japan). To quantify the cell viability, each z‐stack image was separated into green and red channels and converted into a binary image. The numbers of the live cells (*n_green_*) and dead cells (*n_red_*) were counted using the "Spot Detection" tool. Cell viability was determined as

(1) $$\mathrm{Cell}\;\mathrm{viability}=\frac{n_{green}}{n_{green}+n_{red}}100\%$$

### Immunofluorescence Staining of Spheroids

For immunofluorescence staining, the cell spheroids were removed from the microcavities and fixed with 4 wt.% paraformaldehyde (Biosharp) for 30 min. Subsequently, the spheroids were rinsed three times with PBS to remove paraformaldehyde. Then, 0.2% Triton‐X 100 (Sigma‐Aldrich) was added to permeabilize spheroids for 15 min at room temperature. After that, the spheroids were washed with PBS to remove the excess Triton‐X 100 and incubated with 2% bovine serum albumin (BSA, FIYUBIO) for 30 min. For Ki67 staining, Ki‐67 monoclonal antibody Alexa Flour 488 (1:800 dilution, Invitrogen) was added to treat the spheroids for 90 min. For F‐actin staining, Alexa Fluor 555 Phalloidin (1:1000, Yeasen) was diluted in 1% BSA to form the working solution. The spheroids were incubated in the working solution for 90 min. For nuclei staining, 1 µg mL^−1^ of Hoechst 33342 (Thermo Scientific) in PBS was added to treat spheroids for 10 min at room temperature. The stained spheroids were imaged using the confocal microscope. The confocal z‐stack images were acquired and processed with the NIS‐Elements AR 5.01 software.

### Statistical Analysis

Nonlinear regression analysis ([log] inhibitor versus response‐variable slope) of GraphPad Prism 9.0 (GraphPad Software Co., San Diego, USA) was used to determine the IC_50_ values in the drug treatment studies. Synergistic effects of drug combinations were determined by the CompuSyn (ComboSyn Inc., Paramus, USA) and Combenefit software (Cancer Research UK Cambrige Institude, Cambridge, UK). The CI value was calculated by the chou‐talalay method. CI <1, ═1, and >1 indicates synergism, additive and antagonism effects, respectively. Statistical significance was determined using the two‐tailed Student's *t* test unless otherwise mentioned, with the following *p* value considered significant: *p < 0.05; **p < 0.01; ***p < 0.001.

## Conflict of Interest

The authors declare no conflict of interest.

## Author Contributions

Q.F., J.Z.P, J.H., Z.G.C., and Y.X.C. conceived the idea. Y.X.C., J.B.C., and H.F.W. built the instrument. H.F.W. wrote the software. M.T.Z. designed, fabricated, and tested the performance of micropillar arrays. J.H., Z.G.C., and Y.Z. provided clinical samples and participated in the design of drug treatments. Y.X.C., Y. Z., and Y. J. Y. cultured the cells. Y.X.C. planned and performed experiments. Y.X.C. and Q.F. contributed to data analysis and interpretation, as well as figure preparation. Y.X.C. and Q. F. wrote and revised the manuscript. Y.X.C., Y.Z., Y.J. Y., M.T.Z., Y.R.J., J.W., and Q.F. contributed to the supplementary experiments. All authors reviewed and provided comments on the paper. Q.F., J.Z.P., J.H., and Z.G.C. supervised the studies and acquired funding.

## Supporting information



Supporting Information

Supplemental Movie 1

Supplemental Movie 1

Supporting Information

## Data Availability

The data that support the findings of this study are available in the supplementary material of this article.
