# Supplementary material for: Rapid Microfluidic Drug Sensitivity Testing Within 5 Days Using Minimal Clinical Tumor Samples
Source: Adv Sci (Weinh). 2025 Dec 8;13(9):e11065. doi: 10.1002/advs.202511065 (PMC12903994; doi:10.1002/advs.202511065)
Supplement: Supplementary file 1 — Supporting Information [file ADVS-13-e11065-s003.pdf]

Supporting Information

Rapid Microfluidic Drug Sensitivity Testing within 5 Days Using Minimal Clinical Tumor Samples

Yi-Xue Chen[1], Yi Zhang[2,5,6], Yu-Jie Yan[5], Meng-Ting Zhang[1], Jian-Bo Chen[1], Yi-Rong Jiang[1], Jie Wu[1], Hui-Feng Wang[1], Jian-Zhang Pan[1,3*], Zhi-Gang Chen[2,5,6*], Jian Huang[2,5,6*] & Qun Fang[1,3,4*]

[1] Institute of Microanalytical Systems, Department of Chemistry, Zhejiang University, Hangzhou, 310058, China

[2] Department of Breast Surgery (Surgical Oncology), Second Affiliated Hospital, Zhejiang University School of Medicine, Hangzhou, 310009, China

[3] Single-cell Proteomics Research Center, ZJU-Hangzhou Global Scientific and Technological Innovation Center, Hangzhou, 311200, China

[4] Key Laboratory for Biomedical Engineering of Ministry of Education, Cancer Center, Zhejiang University, Hangzhou, 310007, China

[5] Key Laboratory of Tumor Microenvironment and Immune Therapy of Zhejiang Province, Hangzhou, 310052, China

[6] Cancer Centre, Zhejiang University, Hangzhou, Zhejiang, 310058, China

🖂e−mail: fangqun@zju.edu.cn; drhuangjian@zju.edu.cn; chenzhigang@zju.edu.cn; kelvonpan@zju.edu.cn

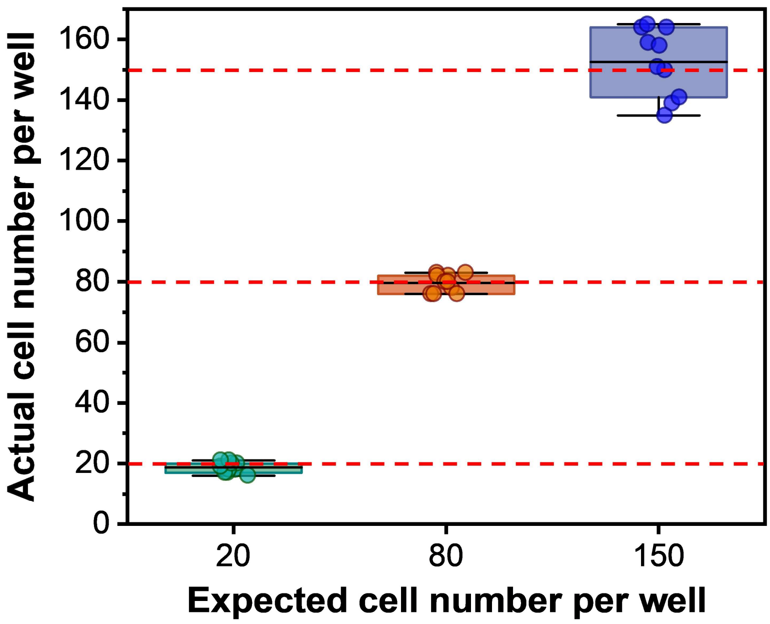

Supplementary Figure 1. Evaluation of the precision and accuracy of the system for cell seeding. The experiment was performed using A549 cells, a 384-well plate, and the SODA system with different expected cell seeding number of 20, 80 and 150 in each well.

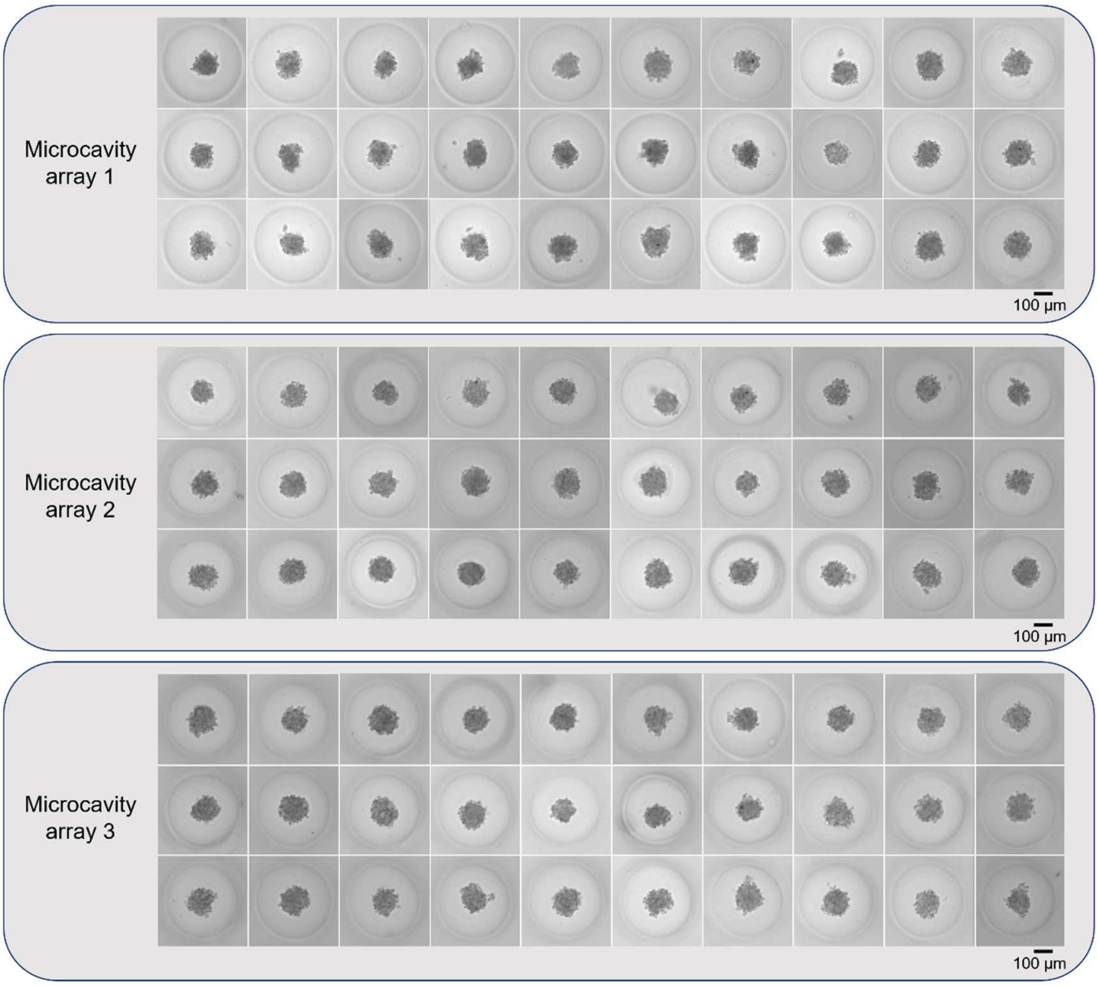

Supplementary Figure 2. Cell spheroids containing 110 cells in three different microcavity arrays after 72 h culture. Scale bars, 100 µm.

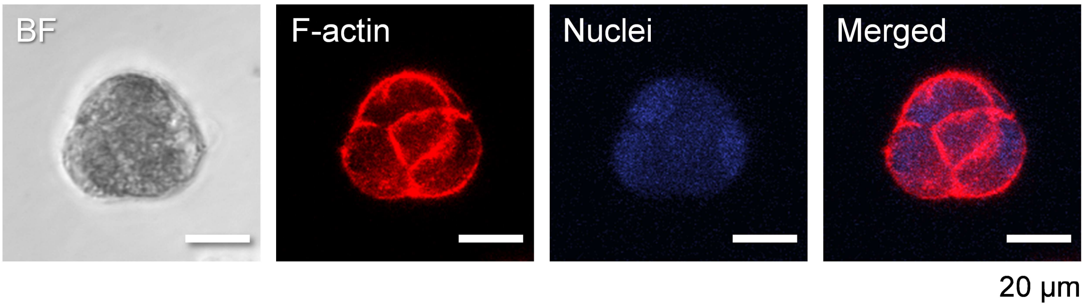

Supplementary Figure 3. Bright field (BF) and immunofluorescence staining images of the spheroids containing an average cell seeding number of 7 cells after 7 d culture, stained by Alexa Fluor 555 Phalloidin (red) for F-actin and Hoechst 33342 (blue) for nuclei. The right-most image shows the merged fluorescence image of the left two fluorescence images.

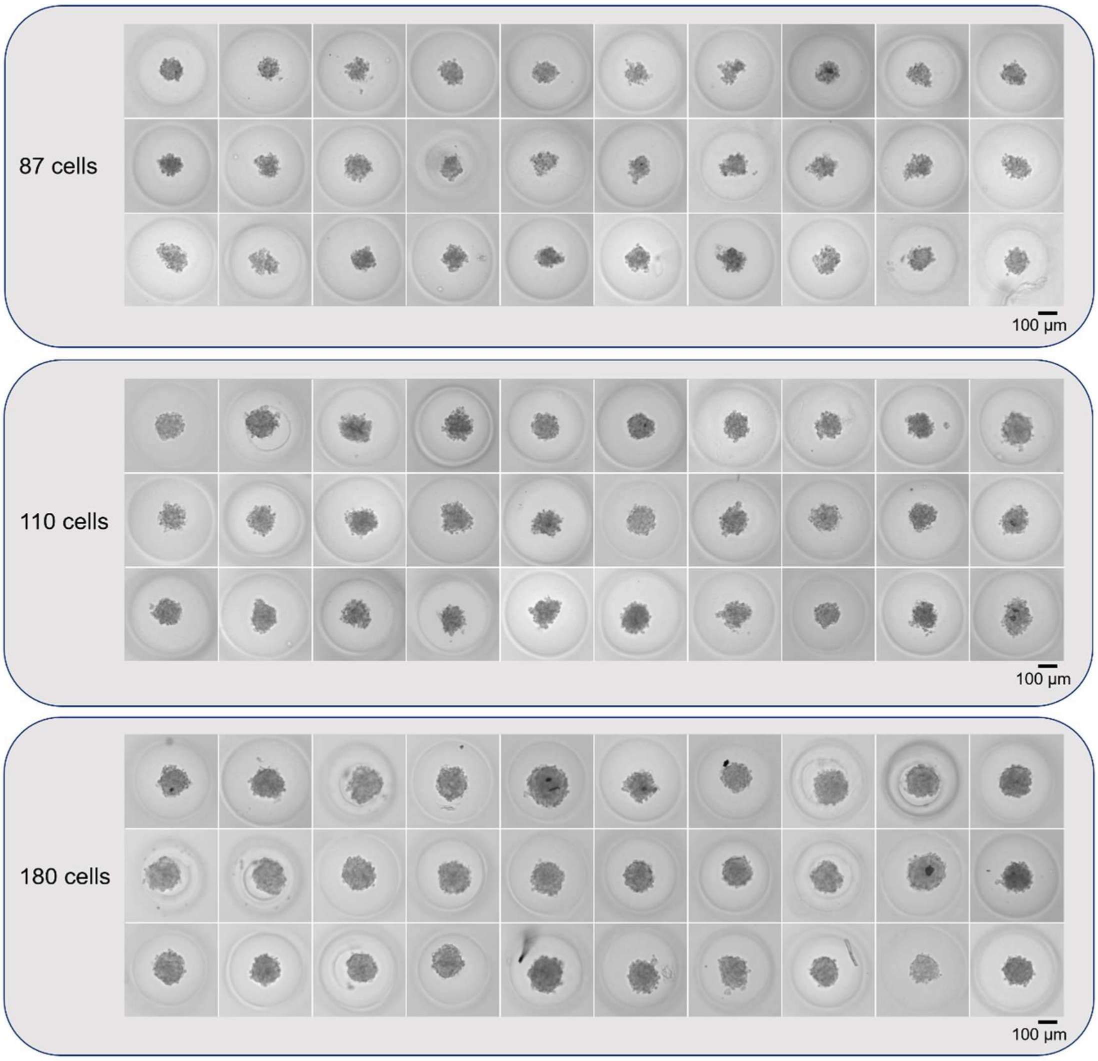

Supplementary Figure 4. Bright field images of HeLa cell spheroids containing different average numbers of cells of 87, 110 and 180 in microcavities after 72 h culture. Scale bars, 100 μm.

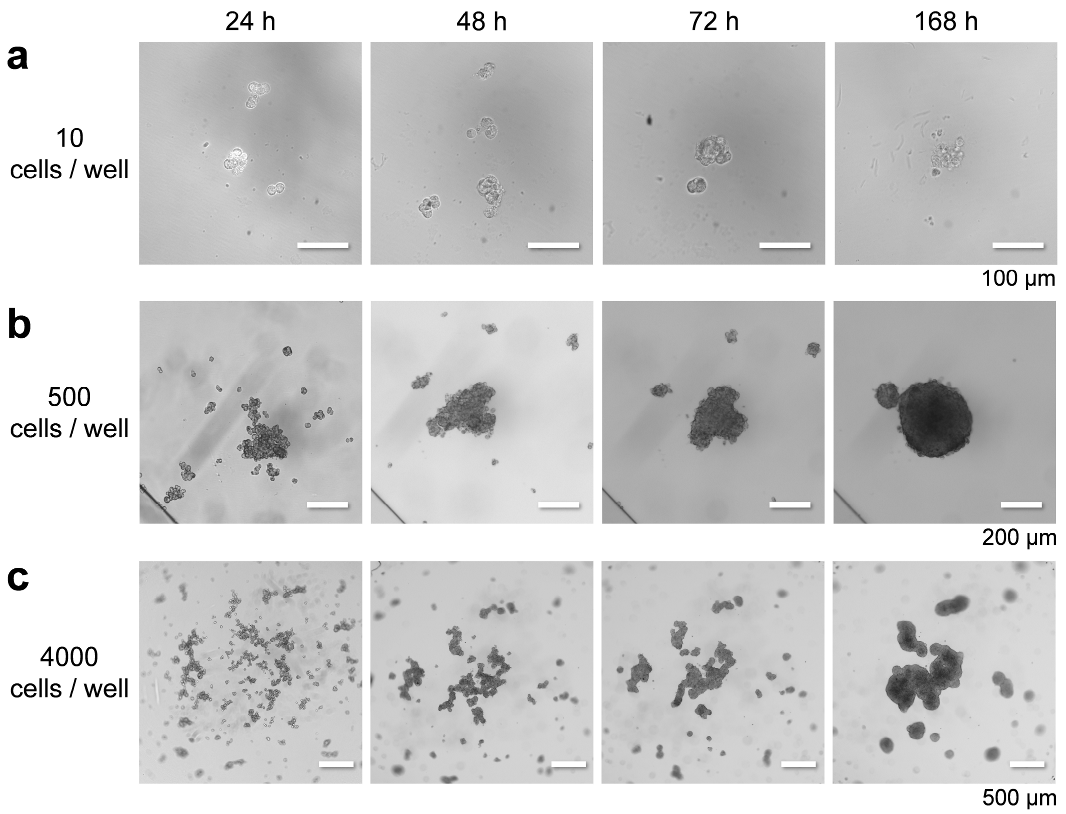

Supplementary Figure 5. Continuous monitoring of the 3D culture process of HepG2 cells with different initial cell densities in the wells of ULA plates during 7 days.

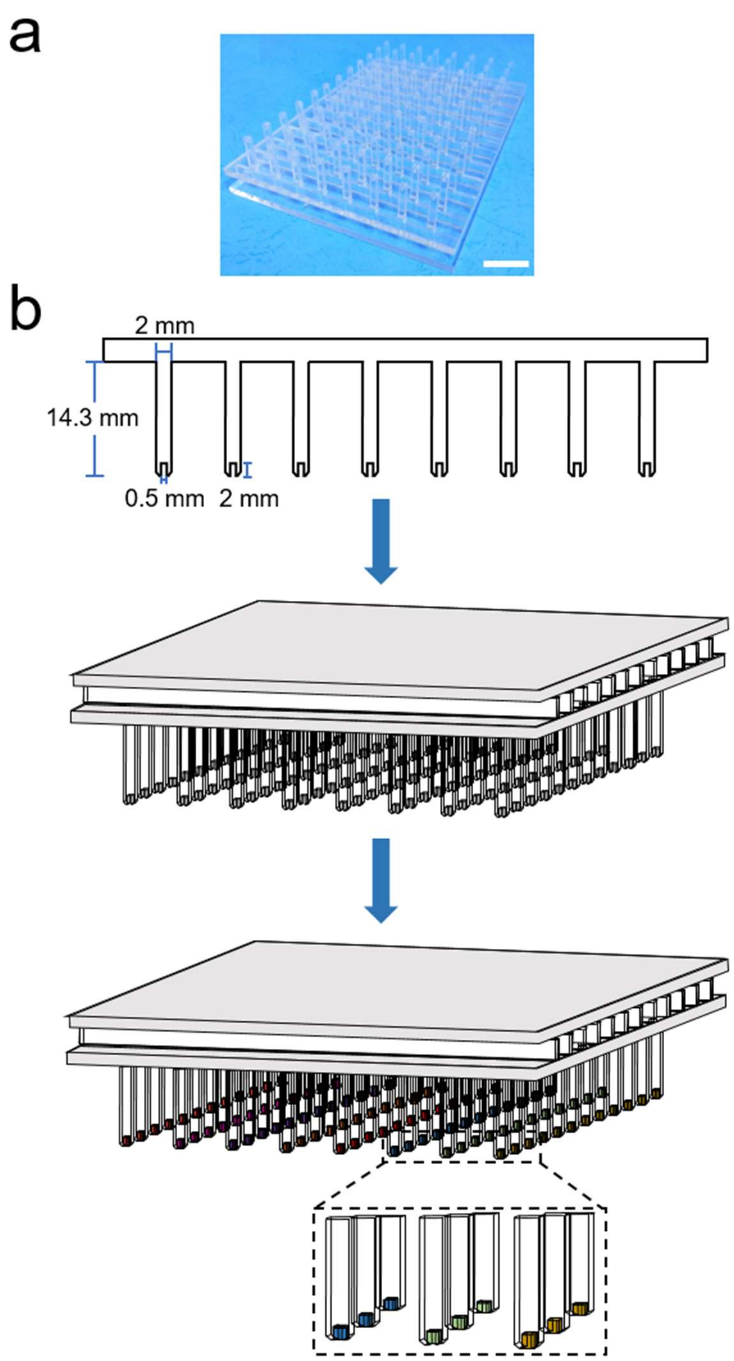

Supplementary Figure 6. The design of the micropillar array. **(a)** The image of a 96-micropillar array. Scale bar, 1 cm. **(b)** Schematic diagrams of the assembling and using of a 96-micropillar array, with different types and concentrations of drug solutions loaded into the micropillar array.

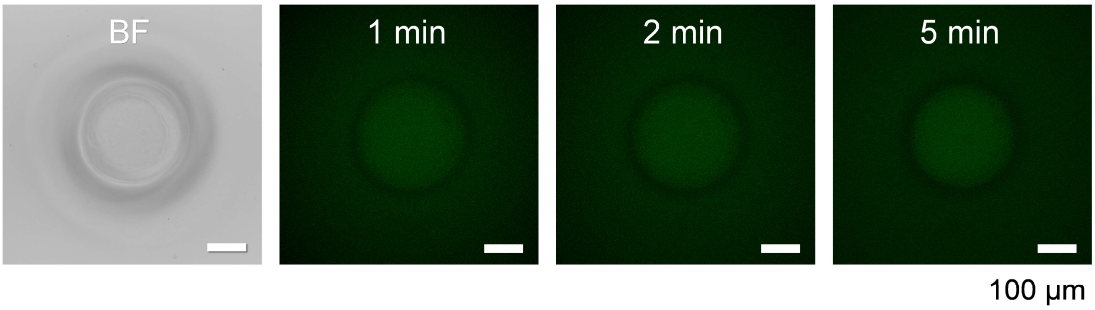

Supplementary Figure 7. Typical images obtained during the monitoring of the diffusion of the fluorescein solution within the hydrogel microcavity over a 5-min period after the solution was added into the microcavity.

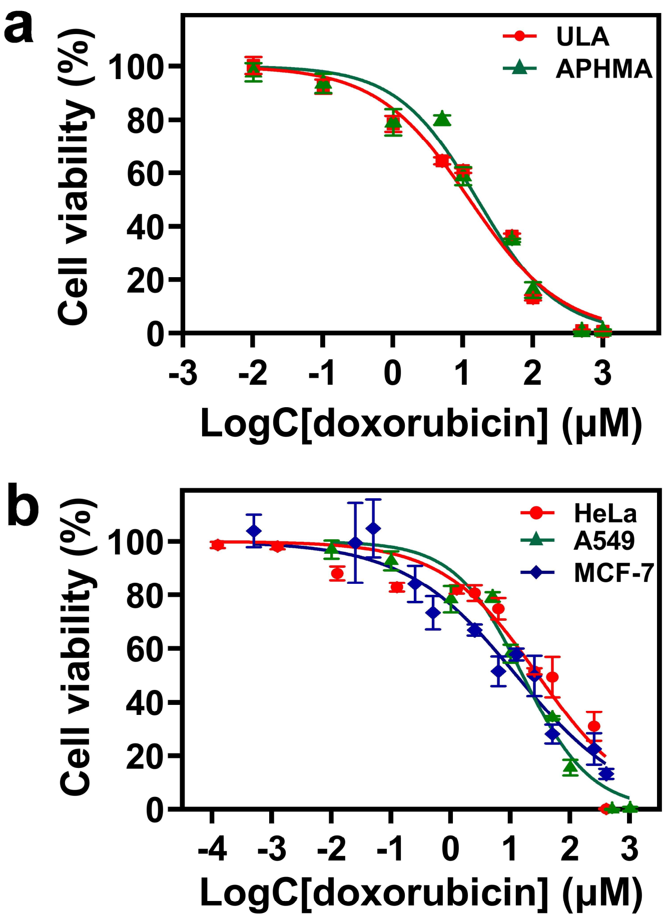

Supplementary Figure 8. (a) Variations of the viabilities of A549 cell spheroids at different concentrations of doxorubicin using the APHMA and ULA plate-based systems. **(b)** Variations of the viabilities of HeLa, A549 and MCF-7 cell spheroids at different concentrations of doxorubicin in the APHMA system.

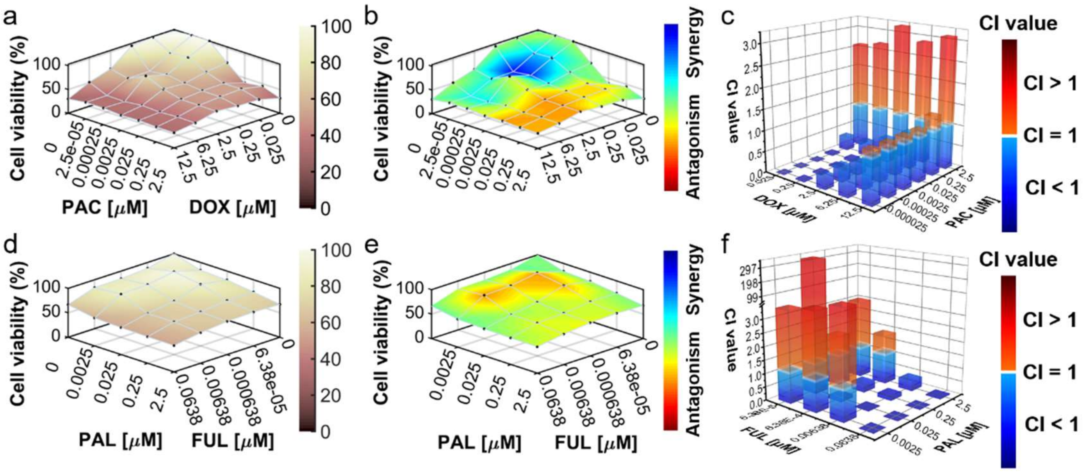

Supplementary Figure 9. Drug sensitivity tests of 2D-cultured MCF-7cells. Cell viabilities of 2D-cultured MCF-7 cells at different doses of doxorubicin + paclitaxel **(a)** and fulvestrant + palbociclib **(d)**. Therapeutic efficacy of 2D-cultured MCF-7 cells with doxorubicin + paclitaxel **(b)** and fulvestrant + palbociclib **(e)** at different doses analyzed by the Bliss model. CI values of the 2D-cultured MCF-7 cells with doxorubicin + paclitaxel **(c)** and fulvestrant + palbociclib **(f)** at different doses calculated by the CompuSyn software. Data are shown as mean ± std. dev for all cells.

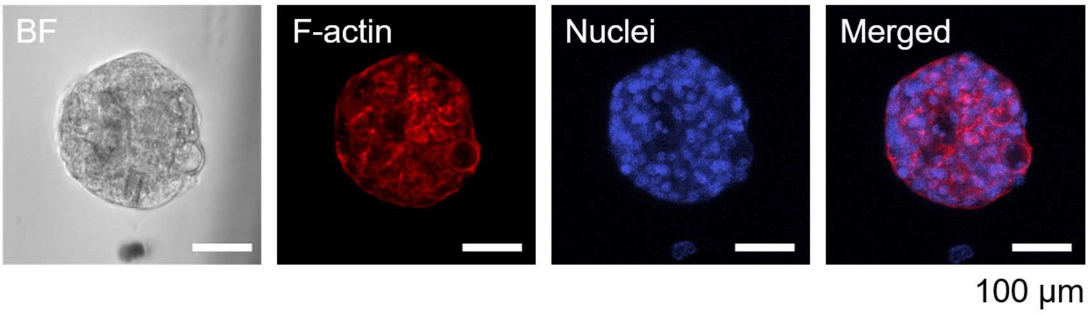

Supplementary Figure 10. Bright field (BF) and immunofluorescence staining images of the patient-derived primary cell spheroid with an average cell seeding number of 200 cells after 7 d culture, stained by Alexa Fluor 555 Phalloidin (red) for F-actin and Hoechst 33342 (blue) for nuclei. The right-most image shows the merged fluorescence image of the left two fluorescence images.

Supplementary Table 1. Combination index (CI) values of HeLa spheroids treated with the combination of doxorubicin (DOX) and cisplatin (CIS) at different doses calculated by the CompuSyn software.

CIS \ DOX	1.25×10^{-3} (μM)	0.125 (μM)	1.25 (μM)	2.5 (μM)	25 (μM)
1.25×10^{-4} (μM)	1.0×10^{9}	0.81	2.1	3.1	5.2×10^{-2}
1.25×10^{-3} (μM)	1.2×10^{6}	2.1	0.75	0.75	9.9×10^{-2}
1.25×10^{-2} (μM)	3.2×10^{23}	3.0	0.43	0.47	0.14
0.125 (μM)	4.4×10^{5}	90	0.67	0.43	0.48
1.25 (μM)	1.7×10^{5}	2.0×10^{2}	0.59	0.11	0.77
2.5 (μM)	2.7×10^{2}	1.9×10^{2}	0.17	8.1×10^{-3}	0.41

Supplementary Table 2. Combination index (CI) values of HeLa tumor spheroids treated with the combination of doxorubicin and sorafenib at different doses calculated by the CompuSyn software.

SOR \ DOX	0.125 (μM)	1.25 (μM)	2.5 (μM)	6.25 (μM)
0.125 (μM)	0.50	0.73	4.5×10^{-2}	9.3×10^{-2}
1.25 (μM)	3.8	0.94	0.20	0.13
2.5 (μM)	1.5	0.91	0.31	0.26
6.25 (μM)	1.7	1.6	0.70	0.14

Supplementary Table 3. Combination index (CI) values of HeLa tumor spheroids treated with the combination of sorafenib and cisplatin at different doses calculated by the CompuSyn software.

SOR / CIS	1.25 (μM)	2.5 (μM)	6.25 (μM)
0.125 (μM)	2.1	3.7×10^{4}	8.2
1.25 (μM)	2.3×10^{2}	10	5.1
2.5 (μM)	1.9	0.94	2.2

Supplementary Table 4. Combination index (CI) values of MCF-7 tumor spheroids treated with the combination of doxorubicin and paclitaxel at different doses calculated by the CompuSyn software.

PAC / DOX	0.25 (μM)	1.25 (μM)	2.5 (μM)	12.5 (μM)	25 (μM)
0.25 (μM)	2.7	1.4	6.5×10^{3}	35	0.11
2.5 (μM)	0.11	9.0×10^{-2}	0.11	0.13	5.9×10^{-2}
6.25 (μM)	0.31	0.28	0.44	0.25	0.15
12.5 (μM)	0.49	0.44	0.42	0.37	0.24
25 (μM)	0.72	0.64	0.74	0.37	0.37

Supplementary Table 5. Combination index (CI) values of MCF-7 tumor spheroids treated with the combination of fulvestrant and palbociclib at different doses calculated by the CompuSyn software.

PAL / FUL	0.025 (μM)	0.25 (μM)	2.5 (μM)
3.19×10^{-3} (μM)	2.5×10^{-3}	0.65	1.0
3.19×10^{-2} (μM)	0.33	5.5×10^{-2}	0.41
0.319 (μM)	2.8×10^{-2}	1.1×10^{-2}	0.60
6.38 (μM)	0.73	1.5	0.74

Supplementary Table 6. Combination index (CI) values of 2D-cultured MCF-7 tumor cells treated with the combination of doxorubicin and paclitaxel at different doses calculated by the CompuSyn software.

PAC / DOX	2.5×10^{-5} (μM)	2.5×10^{-4} (μM)	2.5×10^{-3} (μM)	0.025 (μM)	0.25 (μM)	2.5 (μM)
0.025 (μM)	4.3×10^{-3}	5.1×10^{-3}	7.8×10^{-3}	3.6×10^{-2}	0.34	2.6
0.25 (μM)	3.0×10^{-2}	3.2×10^{-2}	3.4×10^{-2}	6.2×10^{-2}	0.35	2.7
2.5 (μM)	0.30	0.37	0.31	0.31	0.57	3.2
6.25 (μM)	0.60	0.60	0.63	0.63	0.86	2.9
12.5 (μM)	1.2	1.2	1.2	1.2	1.4	3.1

Supplementary Table 7. Combination index (CI) values of 2D-cultured MCF-7 tumor cells treated with the combination of fulvestrant and palbociclib at different doses calculated by CompuSyn software.

PAL / FUL	0.0025 (μM)	0.025 (μM)	0.25 (μM)	2.5 (μM)
6.38×10^{-5}(μM)	3.4×10^{7}	3.3×10^{2}	1.9	3.0
6.38×10^{-4} (μM)	55	0.13	0.31	1.9
6.38×10^{-3} (μM)	1.2×10^{2}	1.9×10^{-2}	0.16	0.40
6.38×10^{-2} (μM)	4.6×10^{-3}	3.2×10^{-3}	6.8×10^{-3}	4.6×10^{-2}

Supplementary Table 8. Clinical information of the patient-derived cell samples tested in this work.

Line	Sex	Menopausal status	Metastatic status	Histology	Tissue origin	Tissue source	ER	ER %	PR	PR %	HER2	HER2 FISH	Prior treatments received
S1	Female	Pre-menopausal	Non-metastatic	IDC	Primary breast	Breast	Negative	0	Negative	0	Negative	Negative	Neoadjuvant chemotherapy: ECT
S2	Female	Menopausal	Non-metastatic	DCIS	Primary breast	Breast	Negative	0	Negative	0	3+	N/A	None
S3	Female	Pre-menopausal	Non-metastatic	IDC	Primary breast	Breast	Negative	0	Negative	0	Negative	N/A	None
S4	Female	Pre-menopausal	Non-metastatic	IDC	Primary breast	Breast	Positive	80+	Positive	60	Negative	N/A	None
S5	Female	Pre-menopausal	Non-metastatic	IDC	Primary breast	Breast	Positive	90+++	Positive	70+++	2+	Positive	None
S6	Female	Pre-menopausal	Non-metastatic	IDC	Primary breast	Breast	N/A	N/A	N/A	N/A	N/A	N/A	None
S7	Female	Pre-menopausal	Non-metastatic	DCIS	Primary breast	Breast	Positive	80+	Negative	0	1+	N/A	None
S8	Female	Menopausal	Non-metastatic	IDC	Primary breast	Breast	Negative	0	Negative	0	2+	Negative	Neoadjuvant chemotherapy: ECT
S9	Female	Menopausal	Non-metastatic	IDC	Primary breast	Breast	Positive	95+++	Positive	80+++	1+	N/A	None

S10	Female	Menopausal	Non-metastatic	IDC	Primary breast	Breast	N/A	N/A	N/A	N/A	N/A	N/A	None
S11	Female	Menopausal	Non-metastatic	IDC	Primary breast	Breast	N/A	N/A	N/A	N/A	N/A	N/A	None
S12-S21	Female	Pre-menopausal	Non-metastatic	Fibroadenoma	Primary breast	Breast	Positive	N/A	N/A	N/A	N/A	N/A	None

Supplementary Table 9. IC_{50} values of different drugs for breast tumor patients. (R means IC_{50} is not available since the viabilities of the cells are >50% under all the concentrations.)

Patients	Tamoxifen (μM)	Doxorubicin (μM)	Fulvestrant (μM)
S1	20.0	/	4.6
S2	32.6	4.0	/
S3	R	3.0	0.6
S4	6.2	0.3	1.3
S5	0.92	85.1	/
S6	0.24	10.3	0.2
S7	0.07	77.2	/
S8	R	69.9	R
S9	2.3	R	/
S10	R	265.2	R
S11	R	162.1	/
S12	0.5	20.8	/
S13	5.6	/	/
S14	169.2	32.5	R
S15	21.2	321.1	/
S16	58.9	46.4	/
S17	11.5	46.3	132.8
S18	50.0	22.4	R
S19	1.1	5.6	25.2
S20	50.0	0.3	2.3
S21	15.9	7.9	R

Supplementary Table 10. Combination index (CI) values of the primary cell sample of Patient S1 treated with the combination of fulvestrant and palbociclib at different doses calculated by the CompuSyn software.

FUL \ PAL	0.025 (μM)	0.125 (μM)	0.25 (μM)
0.025 (μM)	0.16	0.12	0.13
0.125 (μM)	0.56	0.57	0.61
0.25 (μM)	0.98	0.51	0.18
1.25 (μM)	1.67	0.75	0.68

Supplementary Table 11. Combination index (CI) values of the primary cell sample of Patient S3 treated with the combination of fulvestrant and palbociclib at different doses calculated by the CompuSyn software.

PAL / FUL	0.05 (μM)	0.5 (μM)	2.5 (μM)	5 (μM)
6.38×10^{-4} (μM)	3.12×10^{-3}	2.64×10^{-2}	0.118	0.253
6.38×10^{-3} (μM)	4.59×10^{-3}	2.62×10^{-2}	0.125	0.217
6.38×10^{-2} (μM)	2.14×10^{-2}	3.85×10^{-2}	0.122	0.160
0.638 (μM)	1.51	0.163	0.202	0.119

Supplementary Table 12. Combination index (CI) values of the primary cell sample of Patient S4 treated with the combination of fulvestrant and palbociclib at different doses calculated by the CompuSyn software.

PAL / FUL	5×10^{-4} (μM)	5×10^{-3} (μM)	0.5 (μM)
5×10^{-4} (μM)	9.3×10^{2}	4.8×10^{-3}	0.19
5×10^{-3} (μM)	9.3×10^{4}	6.1×10^{-3}	0.15
5×10^{-2} (μM)	2.4×10^{-2}	2.6×10^{-2}	0.14
0.25 (μM)	0.13	0.11	0.17
